# A Review on Blockchain Applications in Operational Technology for Food and Agriculture Critical Infrastructure

**DOI:** 10.3390/foods14020251

**Published:** 2025-01-14

**Authors:** Chengliang Zheng, Xiangzhen Peng, Ziyue Wang, Tianyu Ma, Jiajia Lu, Leiyang Chen, Liang Dong, Long Wang, Xiaohui Cui, Zhidong Shen

**Affiliations:** 1 Key Laboratory of Aerospace Information Security and Trusted Computing, Ministry of Education, School of Cyber Science and Engineering, Wuhan University, Wuhan 430072, China; chengliang@whu.edu.cn (C.Z.); pengxiangzhen@whu.edu.cn (X.P.); ziyuewang@whu.edu.cn (Z.W.); tianyuma@whu.edu.cn (T.M.); jiajialu@whu.edu.cn (J.L.); cly_edu@whu.edu.cn (L.C.); dongliang0607@whu.edu.cn (L.D.); 2 College of Information and Communication, National University of Defense Technology, Changsha 430013, China; wanglong17c@nudt.edu.cn

**Keywords:** food and agriculture, blockchain technology, critical infrastructure, cybersecurity, bibliometric analysis

## Abstract

The food and agriculture sector is a cornerstone of critical infrastructure (CI), underpinning global food security, public health, and economic stability. However, the increasing digitalization and connectivity of operational technologies (OTs) in this sector expose it to significant cybersecurity risks. Blockchain technology (BT) has emerged as a transformative solution for addressing these challenges by enhancing network security, traceability, and system resilience. This study presents a comprehensive review of BT applications in OT security for food and agriculture CI, employing bibliometric and content analysis methods. A total of 124 relevant articles were identified from six databases, including the Web of Science Core Collection and MEDLINE^®^. Bibliometric analysis was conducted across five dimensions: publication year, literature type, journal distribution, country contributions, and keyword trends. The findings are meticulously organized through tables, charts, and graphs. The year 2018 marked a surge in research within this domain, with the IEEE Internet of Things Journal and IEEE ACESS emerging as the most prolific journals, each boasting nine publications. The United States, China, and India are at the forefront in terms of journal citation counts. Our analysis determined that a reference count of 37 serves as an appropriate threshold. Otoum Safa stands out as the author with the highest number of published articles, totaling four. Keywords such as “blockchain”, “internet of things”, “smart contract”, “security”, and “critical infrastructure” appear with significant frequency. The statistics, trends, and insights gleaned from this bibliometric analysis can guide researchers in the OTCI field to forge a coherent and logical research trajectory. Content analysis further identified six key research areas within this domain: identity authentication and data verification, secure access control, attack detection and perception, data security and protection, data backup and recovery, and attack assessment and attribution. Based on these insights, a general framework is proposed to guide future research and practical applications of BT in securing OT within food and agriculture CI. This study systematically analyzes the current research landscape, challenges, and opportunities for BT in securing the OT critical to food and agriculture CI. By bridging the gap between blockchain innovations and the operational needs of the food and agriculture sector, this work contributes to advancing strategic implementation and improving the security of CI systems.

## 1. Introduction

The food and agriculture sector is a critical component of global critical infrastructure (CI), underpinning food security, public health, and economic stability. This sector comprises nearly 1.9 million farms, over 700,000 restaurants, and more than 220,000 registered food manufacturing, processing, and storage facilities in the United States alone, contributing 5.6% to the national GDP and supporting 10.4% of total employment [1]. Beyond its direct economic impact, food and agriculture are intricately connected with other critical infrastructures. For instance, water and wastewater systems provide essential resources for irrigation and food processing, transportation networks facilitate the logistics of agricultural goods and livestock, energy systems power farming equipment and processing plants, and the chemical industry supplies fertilizers and pesticides crucial for crop production [2]. However, the increasing digitalization of this sector exposes it to significant cybersecurity threats. Data breaches, system manipulation, and cyberattacks jeopardize production, processing, and logistics operations. Moreover, biological threats such as pandemics and climate change further exacerbate risks to agricultural productivity and supply chain resilience [3,4].

Operational Technology (OT) refers to the use of hardware and software systems to monitor and control physical processes and equipment [5]. In the food and agriculture sector, OT plays a pivotal role in the CI network, exemplified by enabling precise environmental control in vertical farming systems, optimizing dynamic energy usage in large-scale food processing plants, implementing automated pathogen detection in real-time production lines, enhancing disease surveillance and traceability across supply chains. However, these systems face unique cybersecurity challenges. Unlike traditional IT systems, OT directly interacts with physical processes, making it susceptible to cyber–physical attacks such as advanced persistent threats (APTs) and system manipulations [5,6,7]. To protect OTCI (Operational Technology in Critical Infrastructure) network security, traditional research includes firewalls and intrusion detection systems, virtual private networks, vulnerability management and patch management, data encryption, network monitoring and log analysis, and security training and education [6]. Taking chemical industrial control systems that support the production of fertilizers and pesticides needed for crop cultivation as an example, researchers have improved OTCI network protection capabilities through formal analysis of code security, firmware security detection research, Modbus protocol research, PLC monitoring research, and industrial network architecture improvements [7]. Although the above research has increased OTCI’s network protection capabilities to a certain extent, it cannot provide a trusted environment for CI. OTCI can only defend passively, and the defense cost is high.

Blockchain technology (BT) has emerged as a transformative solution to enhance OT security in the food and agriculture sector. BT is a chain integration technology that combines asymmetric encryption, consensus mechanisms, smart contracts, point-to-point (P2P) information dissemination mechanisms, certificate systems, and distributed storage. Compared with centralized architecture, BT has the characteristics of decentralization, anonymity, non-tampering, and traceability. Customized editing of smart contracts can realize automatic verification, judgment, and decision-making, reducing personnel participation and reducing costs [8]. Through BT, a completely trusted operating environment can be provided for CI. It can provide CI with network protection capabilities, including identity authentication, attack detection, authorized access, and other network protection capabilities, and can jointly defend against attacks faced by CI in the physical world and data world [9,10]. In recent years, researchers have conducted in-depth studies on the application of blockchain in the field of CI network security. In this study, bibliometric analysis and content analysis of relevant studies were conducted, and scholars’ research was comprehensively reviewed.

Up to now, some scholars have provided relevant comments on the application of BT in the field of OTCI, as summarized in Table 1 [11,12,13,14,15]. However, several limitations remain. One major limitation is that most reviews rely solely on content analysis without integrating bibliometric analysis alongside it. Additionally, many reviews focus on a single aspect of the field, fail to offer a comprehensive analysis of OTCI as a whole, and do not incorporate the OT dimension. Furthermore, 2018 marked a significant surge in BT research within the domain of OTCI network security.

To fill this gap, a method combining bibliometric analysis and content analysis was employed to review the latest developments in BT in the field of OTCI. This approach offers several key advantages over the relevant review literature listed in Table 1. A combined approach of bibliometric analysis and content analysis was utilized. While bibliometric analysis provides quantitative data, content analysis offers qualitative insights, enabling a comprehensive understanding of the current status, trends, and correlations in the research field. Through quantitative analysis of publication years, document types, journal publications, countries, authors, and keywords, the research output and academic impact can be assessed. This analysis also assists in identifying the latest research dynamics, selecting appropriate publication channels, discovering potential collaborators, and facilitating academic cooperation and exchange. Key themes, methods, and theoretical frameworks were distilled through content analysis, offering scholars a holistic understanding of the field. Furthermore, knowledge gaps in current research and potential future research directions were analyzed, offering guidance for subsequent studies. A comprehensive review and analysis of BT in the field of CI network security were also conducted, providing researchers with a detailed understanding of existing outcomes in this domain. Given the rapid growth of this field, the analysis effectively reflects the developmental dynamics of BT in CI network security. The specific objectives of this review are to address three primary questions:

- What is the current research status of BT in the OTCI field?
- What are the application challenges of BT in the OTCI field?
- What is the trend of BT in the OTCI field?

The remainder of this article is organized as follows. Section 2 provides the definition of BT in the field of OTCI. Section 3 presents the literature search and screening methods and includes bibliometric analysis. Section 4 offers a review of the application of BT in the field of OTCI and analyzes key issues in the OTCI field. In Section 5, a general framework for BT in the OTCI field is designed. In Section 6, the advantages, challenges, and development trends of BT in the OTCI field are analyzed. Section 7 presents the conclusions.

## 2. Defining Blockchain in Operational Technology Security of the Critical Infrastructure

Operational Technologies (OTs) is the use of hardware and software to monitor and control physical processes, equipment, and infrastructure, performing various tasks from monitoring critical infrastructure (CI) to controlling manufacturing workshop robots. As a key component, CI is an important support for social and economic development. CI refers to systems and assets that are crucial to a country. Once damaged or destroyed, it will cause serious damage to national security, economic lifeline, and the health and safety of citizens [11]. The world is entering a new stage of greater reliance on digitization. The theft of data is also rapidly increasing, providing a new carrier for various malicious behaviors to monitor, manipulate, and extort individuals. The widespread application of artificial intelligence is especially exacerbating the complexity and risks of CI. Cybersecurity refers to the protection of hardware, software, and data within network systems to prevent damage, alteration, or leakage due to accidental or malicious reasons, ensuring the continuous, reliable, and uninterrupted operation of systems and network services [11]. The next generation of interconnectivity is breaking the boundary between the digital and physical worlds. Furthermore, it is essential to note that the realm of CI cybersecurity extends beyond network systems, as network threats have now proliferated to affect physical devices. Countries worldwide have introduced policies and strategies aimed at safeguarding CI. In this context, data on how China, the U.S., Germany, the United Kingdom, and Russia define CI have been compiled, as shown in Table 2 [11], ”√” indicates that the category is included.

**Figure 1:**
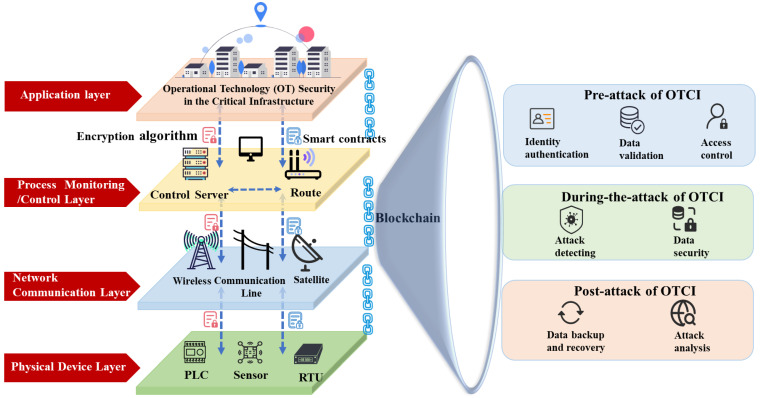
Blockchain diagram in OTCI.

In 2008, Satoshi Nakamoto introduced the concept of “blockchain”, defining it as a chain-like data structure that combines data blocks in chronological order, secured through cryptography to be tamper-resistant and immutable, creating a distributed ledger [16]. Therefore, the blockchain has the characteristics of decentralization, non-tampering, irreversibility, and auditability [17]. In the field of OTCI, BT can provide network protection for the entire CI life cycle. The role of BT in OTCI is shown in Figure 1.

OTCI is divided into the application layer, process monitoring/control layer, network communication layer, and physical device layer. The application layer caters to specific scenarios such as IoT, finance, and transportation. Below lies the process monitoring layer, encompassing control servers and routers. Due to technological advancements, there is a growing prevalence of cloud-based process monitoring systems. These systems connect through the wireless communication layer, including radio and satellite, to physical devices like PLCs and RTUs in the physical device layer. Consequently, they become susceptible to remote hacking attacks, such as network attacks on remote engineering workstations. Moreover, the interconnectivity of devices like PLCs enables attackers to directly launch network attacks on underlying logic components, resulting in severe damage. BT can provide comprehensive network protection throughout the entire lifecycle of OTCI, including identity authentication, data validation, and access control in the pre-attack stage of OTCI. In the during-the-attack stage of OTCI, it can facilitate attack detection. In the post-attack stage of OTCI, it supports data recovery and attack analysis. BT can ensure the security of OTCI at the software, data, network communication, physical equipment, and other levels. OTCI has the characteristics of complex participants, strong dependence on equipment personnel, and a high degree of harm after being attacked. And OTCI lacks identity authentication, access authorization, and other mechanisms. With the increasing interconnectivity of CI, traditional methods have limited effectiveness in addressing the issues faced by OTCI, often leading to problems such as data leakage, tampering, and loss. The development of blockchain, as a significant component of digital transformation, is gaining increasing attention. BT is gradually addressing some of the existing challenges in OTCI.

## 3. Material and Methods

### 3.1. Search Method

In view of the fact that this review considered comprehensive coverage, the historical research data on blockchain in the field of OTCI used in this article were extracted and downloaded from the Web of Science (WoS) of Clarivate Analytics (www.webofscience.com, accessed on 15 September 2023). Since the selected papers only include the Web of Science Core Collection, Chinese Science Citation Database^SM^, SciELO Citation Index, ProQuest™ Dissertations & Theses Citation Index, MEDLINE^®^, and KCI-Korean Journal Database, the above-mentioned database is used as the data source for this review in the article. During the search process, two search methods were designed, as shown below:

- Web of Science was utilized as a search tool. Initially, the keyword “Operational Technology” was used to search the “topic” field (which includes title, abstract, author keywords, and keywords plus) with a publication time cutoff of September 2023, resulting in 76,871 documents. Due to the lack of peer review in preprint databases, the authenticity and reliability of such studies could not be ensured; therefore, preprint documents were excluded. Subsequently, the keyword “Critical Infrastructure” was employed, narrowing the selection to 739 documents. As a distributed ledger technology, BT plays a significant role in safeguarding data within CI. For this review, “Network Security” was used as the third keyword for a refined search, resulting in 106 documents. Finally, “Blockchain” was applied as the fourth keyword for targeted searches, identifying a total of 5 documents, which were included in the Web of Science Core Collection database, covering the period from 2018 to 2023;
- To ensure the quality of paper retrieval, the preprint database was excluded due to the lack of peer-reviewed validation. The keyword “Critical Infrastructure” was then applied, resulting in 43,594 documents, among which 35,006 were included in the Web of Science Core Collection. Additional papers were distributed across other databases, including 704 in the Chinese Science Citation Database^SM^, 292 in the SciELO Citation Index, 6374 in the ProQuest™ Dissertations & Theses Citation Index, 5849 in MEDLINE^®^, and 861 in the KCI-Korean Journal Database. The keyword “Network Security” was subsequently utilized for a refined search, narrowing the selection to 3733 documents. Finally, “Blockchain” was used for targeted searches, identifying a total of 160 documents spanning the years 2017 to 2023.

**Figure 2:**
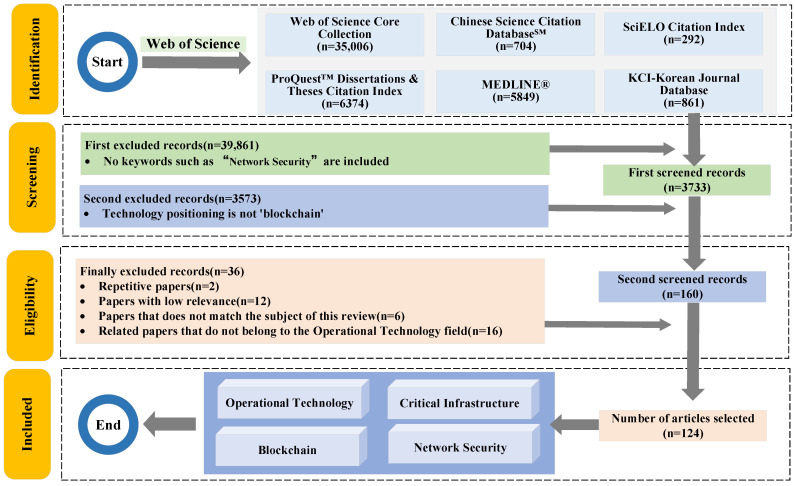
Literature retrieval and selection strategies.

Compared with the two search methods, the first one has more screening rounds than the second one. But focusing on BT, the screening effect is not obvious. The second focuses on BT over a longer time span than the first, with a larger selection of articles but with less focus on the OT field. Considering that CI is a subset of OT in the defined scope, this paper adopts the second retrieval method as the main method and the first retrieval as the auxiliary method for literature screening, as shown in Figure 2.

During the initial screening process, three keywords—“Critical Infrastructure”, “Network Security”, and “Blockchain”—were selected to define the scope of this review. Preprints, which have not undergone peer review, and patents, which present issues consistent with the originality required for academic research, were excluded to ensure the integrity and innovativeness of the reviewed literature. A total of 160 documents were identified. Among these, 2 were duplicate papers, 12 were deemed to have low relevance to this review, and 6 were unrelated to the subject matter, necessitating further refinement. Compared to the initially designed screening method, the adopted approach lacked a precise focus on the field of Operational Technology (OT). Since Critical Infrastructure (CI) defines the research domain and OT serves as its operational mechanism, the impact of “Operational Technology” as a focus was relatively minor for research papers but more significant for review literature. To address this, the initial retrieval method was combined with the current approach, narrowing down relevant review papers within the OT domain and excluding 16 non-review papers in this field. Ultimately, 124 documents were selected, with their subject areas identified as lying at the intersection of “Operational Technology”, “Critical Infrastructure”, “Network Security”, and “Blockchain”.

### 3.2. Review Steps

**Figure 3:**
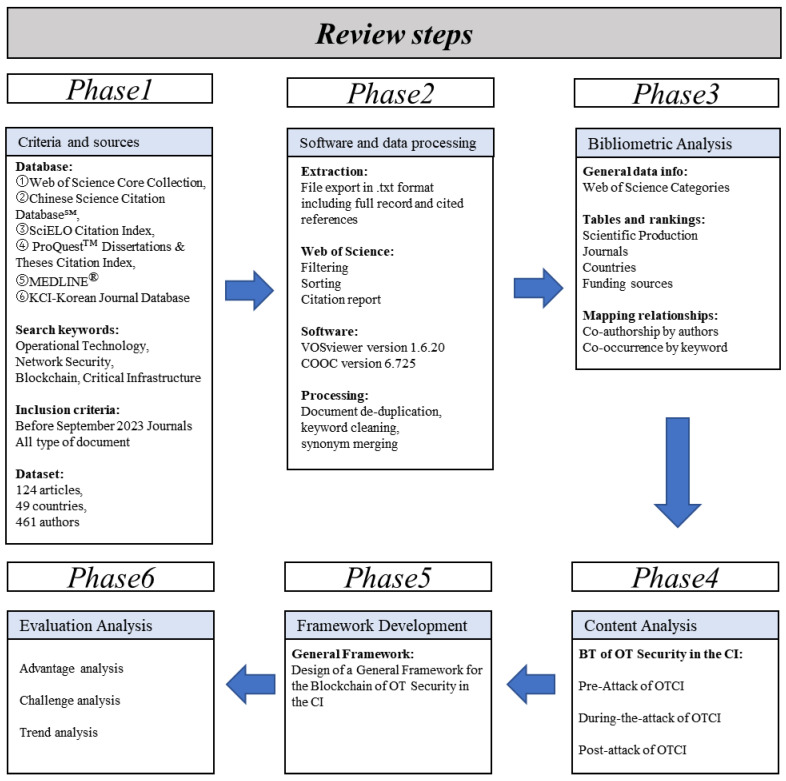
Review steps.

This review study is divided into six systematic phases, as shown in Figure 3. In the first phase, a dataset was obtained through a search strategy, resulting in the selection of 124 articles. In the second phase, the selected literature was exported from the Web of Science, and the exported data were processed by removing duplicates. Using COOC software (version 6.725), keyword cleaning and synonym merging were performed. In the third phase, bibliometric analysis was conducted on the processed data, covering various aspects such as the distribution of publication years, types of literature, publishing journals, and countries of publication [18]. Subsequently, in-depth bibliometric analysis was carried out using VOSviewer software (version 1.6.20), which included constructing an author co-authorship network and a keyword co-occurrence network. In the fourth phase, content analysis was conducted, focusing on the application of blockchain in the security of OTCI. The analysis was divided into three scenarios: pre-attack, during-attack, and post-attack, with a detailed discussion of its impact on the food and agriculture sector. In the fifth phase, a comprehensive research framework was developed, providing valuable insights into the study of blockchain in the field of OTCI. Finally, the current research status and challenges of blockchain in the OTCI field, particularly in the food and agriculture sector, were evaluated, and future research trends were explored.

### 3.3. Bibliometric Analysis

#### 3.3.1. Year of Publication

**Figure 4:**
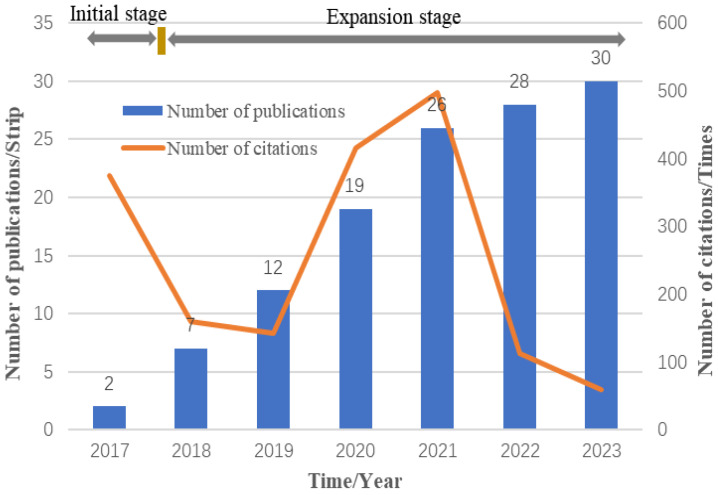
Year of publication.

Figure 4 shows the publication trends of BT in the OTCI field, reflecting the growing interest and development in this area over time. To better understand these trends, it is important to consider key background factors influencing research in this field, such as technological milestones and regulatory developments.

From 2017 to 2023, the number of BT-related publications in the OTCI field can be divided into two distinct phases: the initial phase (2017–2018) and the expansion phase (2019–2023). During the initial phase, the number of publications was relatively low (2 papers in 2017, 7 papers in 2018), but the citation counts were significantly higher (375 citations in 2017, 159 citations in 2018). This early surge in citations can be attributed to the growing interest in BT as a promising technology, driven by major technological advancements, such as the widespread adoption of Ethereum supporting smart contracts, improvements in the performance of blockchain consensus mechanisms, and better computational power for OTCI applications. Additionally, during this period, regulatory bodies began exploring frameworks for the safe and ethical use of emerging technologies like BT, sparking interest and discussions within the academic community. Starting in 2019, the field entered an expansion phase, characterized by a sharp increase in the number of publications, rising from 12 papers in 2019 to 30 papers in 2023. This growth was likely driven by several factors, including further technological advancements, the increasing commercialization of BT applications, and clearer regulatory guidelines for their implementation. As regulations became more defined, particularly in areas such as data privacy, safety standards, and ethical concerns, the research field saw a greater push for innovation and application in OTCI systems. The regulatory environment played a positive role in encouraging both academic exploration and industrial adoption of BT technologies. However, despite the increased number of publications, the citation count peaked in 2021 (498 citations) and gradually declined in the following years. This could be partly because many key studies published earlier in the field have already been widely cited, and newer papers may take time to gain similar recognition. Additionally, since our data collection was limited to September 2023, the downward trend in citations compared to 2021 could be related to the timing of the studies included.

In summary, the publication trends of BT in the OTCI field, as shown in Figure 4, indicate that the technology has gained significant attention in recent years. The increasing recognition from journal publishers, experts, and reviewers reflects advancements in technology and a more stable and favorable environment for research in this field.

#### 3.3.2. Literature Type and Publication Journal

**Figure 5:**
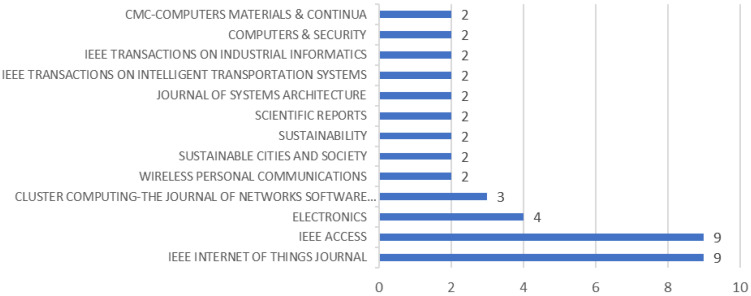
Statistical chart of published journals.

Among the 124 papers selected, there were 119 research papers and 5 reviews. After review, in addition to 3 dissertations, a total of 83 journal papers were reviewed, accounting for 67%. There were 38 conference papers, accounting for 31%. Relevant research on the application of BT in the OTCI field is mostly presented in journals. Among the 83 journal articles, their publication sources came from four databases. They are Web of Science Core Collection, ProQuest™ Dissertation and Dissertation Citation Index Database, Chinese Science Citation Database^SM^, and MEDLINE^®^. These 83 journal articles come from 53 publishing units. Among them, 40 units published 1 journal article, and 9 units published 2 papers. IEEE ACCESS and IEEE INTERNET OF THINGS JOURNAL have the largest number of publications, each publishing 9 papers. Related publications cover many fields, such as power, energy, communications, security, and civil engineering, fully demonstrating the research potential and application prospects of CI security. As shown in Figure 5, these are the units that have published more than two papers. Details of the journal publications (impact factor, h-index, WoS quartile, subject area, etc.) can be found in Appendix A. The data in Appendix A highlight that these journals span multiple disciplinary fields, including computer science, engineering, medicine, and environmental science. Notably, journals with high-impact factors and h-indices are predominantly concentrated in the domains of computer science and engineering. For instance, the IEEE Internet of Things Journal boasts a notable impact factor of 8.2 and an h-index of 47, securing its position in the Q1 category. Similarly, the IEEE Transactions on Industrial Informatics demonstrates an even higher impact factor of 11.7, underscoring its significant influence in the engineering field. Furthermore, interdisciplinary journals, such as Scientific Reports, also showcase impressive metrics, with an impact factor of 3.8 and an h-index of 149. Overall, the table provides a comprehensive overview of the academic impact of these journals across various research fields, serving as a valuable reference for researchers in selecting suitable venues for submission. In addition, it can be pointed out that although many journals have only published one paper in the relevant field, the large number of published journals indicates that the introduction of BT in OTCI is gradually being recognized by publishing houses.

#### 3.3.3. Country

To understand the global distribution and research trends of BT in OTCI and evaluate potential international cooperation opportunities, a statistical analysis was conducted on the countries represented in 124 selected articles. Based on the locations of the authors, 49 countries or regions were identified worldwide, with the top 10 contributing countries presented in Table 3. The number of references helps assess whether scholars from a particular country or region have conducted extensive literature reviews in their scientific research. This contributes to ensuring the depth and breadth of the study, making the results more comprehensive and reliable. It is often used as an indicator to evaluate research quality. From Table 3, it can be observed that among the top 10 contributing countries, the average number of references per paper is 27.97 for the United States, 41.23 for China, 45.42 for India, 44.83 for Saudi Arabia, 48.55 for Pakistan, 51.89 for the United Kingdom, 22.11 for Canada, 32.88 for Australia, 41.43 for the United Arab Emirates, and 29 for Singapore. The average number of references per paper for the top 10 contributing countries is 37.19. This indicates that the United Kingdom, Saudi Arabia, India, the United Arab Emirates, and China extensively referred to previous research when writing their papers. Based on this, we have established that a reference count of 37 is a reasonable threshold, providing researchers with a reference point. The United States is the country with the largest number of references, with a total of 30 articles cited 347 times, of which the most cited single article is 52 times. China and India have a leading position in the citation count of the literature in this dataset, indicating that these two countries have an advantage in the completeness of the literature references. In contrast, developed Western countries such as the U.K., despite having a small number of references, also demonstrate a certain level of competitiveness in terms of citation count. For example, although the U.K. only contributed 9 articles, its total citation count exceeded 500. The low citation numbers in the Middle East and emerging countries indicate that there is room for improvement in the breadth or depth of reference sources in their research literature. It is worth noting that articles published in the IEEE IoT Journal are the most cited literature in this field, with 361 citations [8].

Regarding funding agencies, based on the institutional statistics function of the Web of Science database, a total of 108 funding agencies supported the 124 reviewed publications. The funding sources demonstrate a broad international scope and diversity, primarily concentrated among major research foundations in China, the United States, and Europe. China stands out as the primary funding source, with the “NATIONAL NATURAL SCIENCE FOUNDATION OF CHINA” supporting 11 publications, ranking first. Other Chinese funding agencies, such as the “NATIONAL KEY RESEARCH DEVELOPMENT PROGRAM OF CHINA”, “FUNDAMENTAL RESEARCH FUNDS FOR THE CENTRAL UNIVERSITIES”, and regional funds (e.g., Guangdong Province and Guizhou Province), highlight the comprehensive support provided by the Chinese government for scientific research. Among European and American institutions, the “EUROPEAN UNION (EU)” ranks second, supporting six publications, reflecting its emphasis on international research collaboration. The “UNITED STATES DEPARTMENT OF ENERGY (DOE)” and the “NATIONAL SCIENCE FOUNDATION (NSF)” funded four and three publications, respectively, further showcasing the United States’ focus on technological innovation. Additionally, the “ENGINEERING PHYSICAL SCIENCES RESEARCH COUNCIL (EPSRC)” and the “NATURAL SCIENCES AND ENGINEERING RESEARCH COUNCIL OF CANADA (NSERC)” are also listed as funding sources. Notably, funding from the Middle East and other Asian countries has gradually shown its influence. For instance, Saudi Arabia’s “KING SAUD UNIVERSITY” and South Korea’s “NATIONAL RESEARCH FOUNDATION OF KOREA (NRF)” each supported two studies. Overall, the funding sources reflect the strengthening of international research collaboration, as well as the focused investment and priorities of research institutions in various countries across different technological fields.

#### 3.3.4. Author

**Figure 6:**
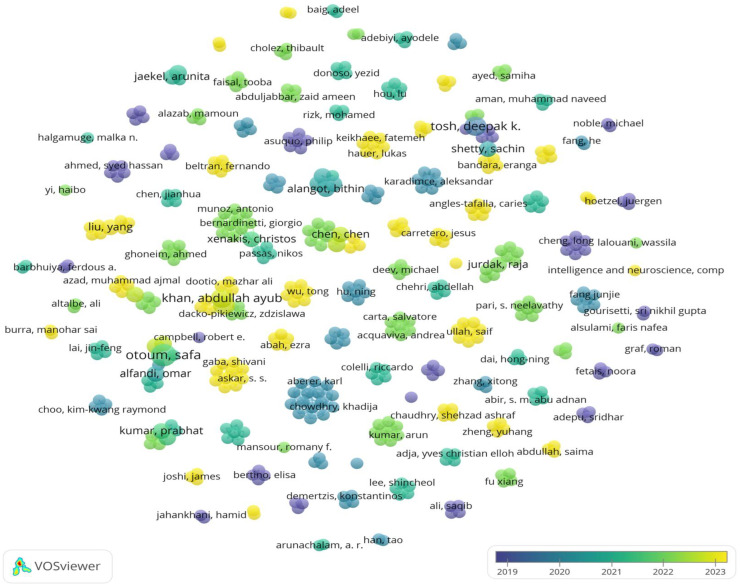
Author co-authorship network diagram.

Tracking the publications of relevant scholars or groups in the field of research is an important way to understand and learn about relevant trends in the field. A total of 461 authors were counted in the selected articles, and they jointly completed 124 papers. A co-authorship analysis of authors was conducted using VOSviewer software, as illustrated in Figure 6. Each circle in the figure represents an author. The different colors represent the specific years of classification of papers published. As can be seen from Figure 6, most research activities in this field are carried out by research teams, and only a few scholars carry out scientific research activities alone. It can be clearly seen that green and yellow circles occupy most of them, indicating that there has been relatively more research in this field in the past two years. Otoum Safa is the author with the highest number of publications (4 papers), but the citation count is relatively low (138 citations), and the total link strength is weak (7). The authors with the highest citation counts are Asuquo Philip, Cao Yue, Cruickshank Haitham, Lei Ao, Ogah Chibueze P. Anyigor, and Sun Zhili (all with 384 citations), but their number of publications is low (only 1 paper each), and their total link strength is also weak (all with a value of 5). Authors with a total link strength of 14 demonstrate a high frequency of collaboration, but each of them has only 1 paper and 79 citations. Khan Abdullah Ayub and Laghari Asif Ali, with relatively high citation counts (170 citations), have also published more papers (3 each), and their total link strength in the collaboration network is higher (12), indicating their greater academic influence. A total of 461 authors were grouped into 100 clusters, indicating relatively weak collaboration among scholars, which requires further strengthening. Research on the application of BT in the field of CI cyber security shows a trend of continued growth and diversification.

#### 3.3.5. Keywords

**Figure 7:**
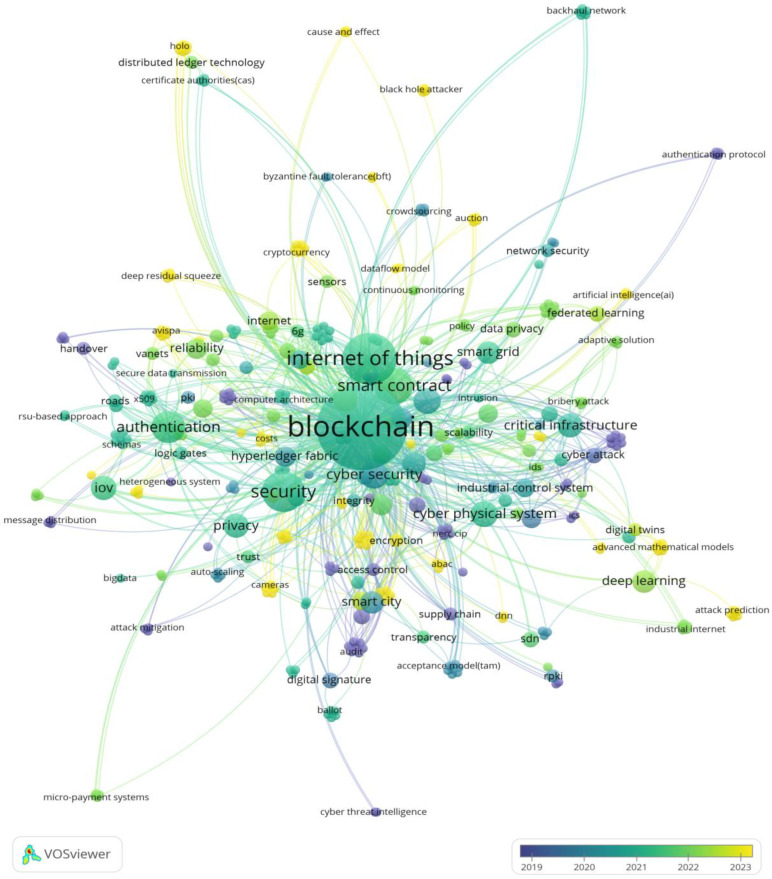
Keyword co-occurrence network diagram.

VOSviewer software was used to conduct keyword analysis on the 124 papers selected, as shown in Figure 7. To better capture the definition of keywords, a joint analysis of the abstracts, titles, and keywords of the selected literature was conducted to broaden the scope of data analysis. Each keyword is represented by a circle. The larger the circle, the higher the frequency of occurrence. Different colors represent different years, and the arc between the circles represents the connection between the keywords. As can be seen from the figure, the circles of keywords such as “blockchain”, “internet of things”, “smart contract”, “security”, and “critical infrastructure” are relatively large. In these 124 articles, the main research focus is the use of BT to solve security problems in CI. In addition, terms such as “authentication”, “edge computing”, “deep learning”, “smart city”, “IoV”, and “industrial control system” also have a high occurrence rate, which shows that OTCI research spans fields such as the internet of things, internet of vehicles, edge computing, artificial intelligence, smart city, and related technologies. These technologies also play an important role in supply chain management, equipment certification, and data tracing in the fields of food and agriculture. Research trends show that blockchain is gradually integrating with artificial intelligence (such as deep learning and federated learning) and emerging technologies (such as “digital twins”) to optimize distributed data management, enhance privacy protection, and network security for CI. In addition, the application of IoT technology in agricultural equipment and sensors provides support for BT in precision agriculture and smart farms.

## 4. Blockchain of Operational Technology Security in the Critical Infrastructure Topic Analysis

**Figure 8:**
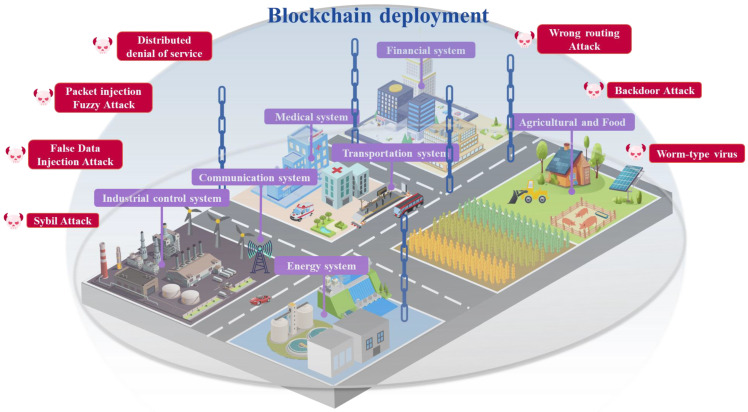
Schematic diagram of BT protection function in OTCI.

Scholars have deeply explored BT’s network security protection in the OTCI field. The protective effect of blockchain is shown in Figure 8. Through cryptographic algorithms, consensus mechanisms, P2P communication mechanisms, smart contracts, certificate signature systems, and decentralized architectures, the deployment of blockchain can defend against various network attacks that CI is susceptible to, including Distributed Denial of Service (DDoS), Packet Injection Fuzzy Attack, False Data Injection Attack, Sybil Attack, Wrong Routing Attack, Backdoor Attack, and Worm-type Virus. A total of 124 research papers related to blockchain in OTCI were identified. Among these, 119 documents (excluding five review papers) were primarily analyzed, as shown in Table 4. This analysis categorizes the research according to the operational phases, which include the pre-attack, during-the-attack, and post-attack stages of OTCI. In the pre-attack stage of OTCI, it comprises two main areas of focus: identity authentication and data validation (38 papers) and secure access control (18 papers). In the during-the-attack stage of OTCI, there are two primary categories: attack detection and perception (24 papers) and data security and protection (30 papers). In the post-attack stage of OTCI, the research is divided into data backup and recovery (four papers) and attack assessment and attribution (five papers).

### 4.1. Pre-Attack of OTCI

#### 4.1.1. Identity Authentication and Data Validation

Identity authentication and data verification can perform boundary defense against attacks and improve OTCI’s network security protection capabilities. Scholars have conducted the following investigations into the application of BT in OTCI identity authentication and data validation.

i. Identity Authentication

BT, as a form of distributed ledger technology, possesses unique characteristics that can ensure the credibility of identity authentication. In the OTCI domain, researchers have explored the use of BT for identity authentication of operators, machines, and other entities to enhance the boundary defense against viruses. Research primarily focuses on OTCI sectors such as communication systems, energy systems, transportation systems, industrial control systems, social media and online communities, and healthcare system security, as shown in Table 5.

BT, as a distributed technology infrastructure, can provide decentralization, anonymity, and an immutable storage and transaction mechanism for the CI sector. It ensures the security and trustworthiness of certificate data. For instance, in blockchain-based identity authentication, a user’s identity is typically represented by a unique cryptographic key pair. When a user seeks to authenticate their identity, they can use their private key to provide a digital signature. The recipient can then use the user’s public key to verify this signature, thereby confirming the authenticity of the user. Furthermore, since the entire identity authentication process takes place within the blockchain, processes such as certificate issuance, verification, and querying are likewise tamper-proof. Compared to traditional identity authentication systems, blockchain-based identity authentication can address issues such as identity forgery, certificate loss, and complex authentication. In the field of communication system security, current research primarily focuses on two directions: the internet of things and the internet. This research is mainly aimed at reducing authentication costs, improving authentication efficiency, and enhancing security. In the context of IoT, scholars have explored the use of BT in identity registration, on-chain storage, block signature verification, and identity verification of senders and receivers within IoT infrastructure domains such as Cyber–Physical Systems (CPSs) [19], Low-Power Wide-Area Networks (LPWANs) [29]. These efforts aim to combat data packet replay and man-in-the-middle attacks, ultimately enhancing security [37]. In the context of IIoT, scholars have explored certificate systems based on BT. These systems are used to establish digital identities for connected devices within IIoT platforms, facilitating identity verification of participating entities [31,32,33]. In the context of future IoT systems, Fang et al. [36] have proposed a multidimensional adaptive solution, leveraging BT as an intelligent process to learn and track all available physical attributes. This approach aims to enhance the reliability and robustness of authentication by integrating multiple attributes. In the context of the internet, scholars’ research primarily focuses on the Domain Name System (DNS). Hu et al. [34] introduced a novel DNS data plane decentralized architecture called Blockzone. This architecture aims to enhance the efficiency of domain name resolution and verification, thereby strengthening DNS’s ability to withstand DDoS and cache poisoning attacks. In the field of energy system security, research primarily focuses on using BT to ensure system resilience, reduce penetration rates, and enhance the security of smart grid operations and management. For instance, Noble et al. [20] designed a location-based blockchain authentication protocol for intelligent grids with high penetration of Distributed Energy Resources (DERs). Singh et al. [23] implemented session key and authentication mechanisms for secure communication. Samy et al. [24] utilized blockchain for user authentication within the network to ensure system resilience and prevent attackers from manipulating relevant smart devices in the smart grid. In the field of transportation system security, scholars primarily focus on research related to the Vehicular Ad Hoc Network (VANET) or vehicle-to-vehicle communication in the context of the internet of vehicles (IoV). Due to the mobility of vehicles, scholars have investigated security aspects within VANETs. Research in this area often includes exploring concepts like secure ownership transfer between infrastructure, physical unclonable functions, and enabling rapid re-authentication of vehicles, as well as real-time sharing of identity authentication results [9,26,28]. In addition, Karim et al. [30] proposed and evaluated a Secure Data Exchange (BSDCE-IoV) scheme based on elliptic curve cryptography for secure communication. This scheme establishes authenticated key protocols between vehicles within each driving area and roadside units (RSUs). Namane et al. [25] introduced a Vehicle Detector Authentication Scheme (VDAS), which permits the computation of the number of sensor nodes for detecting vehicles and performs authentication. In the field of industrial control system (ICS) security, traditional communication protocols in Industrial Control Systems and Supervisory Control and Data Acquisition (ICS/SCADA) systems often lack sufficiently secure mechanisms for providing device authentication. Rivera et al. [22] presented an advanced decentralized SCADA system architecture that leverages the advantages of tamper-resistant ledgers for authentication purposes. Gomez et al. [27] introduced a blockchain-based SRAM PUF (Physical Unclonable Function) authentication and integrity protocol. This protocol aims to ensure continuous identity authentication of field sensors and provides robust data stream integrity verification by utilizing distributed ledgers and hardware security primitives. In the field of social media and online community security, Prodan et al. [21] introduced the ARTICONF project, which offers identity management for users participating in the network. It allows users to couple their digital identities with their real-world identities within the local environment. In the field of healthcare system security, Nyangaresi et al. [35] have developed a verifiable secure and privacy-preserving configuration protocol. This protocol facilitates mutual authentication between biometric sensor units and hospital medical servers, establishing secure communication sessions for subsequent data exchange. The above research highlights that in the context of the food and agriculture sector, cyberattacks may lead to supply chain disruptions, impacting food supply and prices while undermining consumer trust in food brands. Moreover, disruptions to the stability of energy systems can affect the operation of food processing and agricultural machinery, while delays or interruptions in logistics and distribution may result in food waste and decreased supply chain efficiency. Data tampering in industrial control systems poses threats to food safety and production efficiency, and attacks on social media can influence consumer purchasing decisions. Additionally, cyberattacks on healthcare systems may indirectly impact the health and safety of food processing workers, thereby affecting the productivity of the food and agriculture sector.

The above-mentioned research investigates the application of BT in various areas of OTCI for identity authentication. These efforts contribute to the development of secure identity authentication mechanisms, thereby enhancing the network security and resilience of CI. However, using blockchain for identity authentication can introduce challenges such as increased latency, resource consumption, and higher communication requirements compared to traditional mechanisms. In real-world application scenarios, there still exists a gap when dealing with large-scale and high real-time identity authentication demands.

ii. Data Validation

Data validation plays a fundamental role in the cybersecurity defense of OTCI networks. It serves as a foundational layer for verification and security. Traditional data validation processes are often manual and predominantly centralized in their storage, which can lead to challenges in ensuring the authenticity of data validation. Some network viruses, such as worm viruses, trojan viruses, macro viruses, and others, have the capability to manipulate, contaminate, or delete data within CI, posing significant security risks. Indeed, BT unquestionably enables automated data validation, ensuring the security of data [51]. Research on data validation by scholars primarily focuses on OTCI areas such as communication systems, energy systems, transportation systems, industrial control systems, financial systems, and building facilities, as shown in Table 6.

In the field of communication system security, research primarily focuses on using BT to perform data validation in communications between IoT devices [48,54]. For example, Martinez et al. [46] proposed a method for Continuous Delivery/Continuous Verification (CD/CV) of IoT data streams in the context of the edge-fog-cloud using blockchain smart contracts. Regarding data auditing, Burra et al. [38] designed an efficient and secure distributed and decentralized network setup based on certificateless cryptography for shared group data auditing. This approach mitigates security issues resulting from the compromise of key generation centers. Furthermore, scholars have explored the continuous verification of identity information using BT [53]. In the field of energy system security, researchers have highlighted that blockchain can address concerns related to security, privacy, identity, and the immutability and verifiability of records. It enables end-user devices to securely share their energy resources within network environments [42,44]. In the field of transportation system security, researchers primarily investigate the use of BT for data validation, supporting identity authentication for vehicles and individuals. This research also focuses on maintaining and authenticating shared data to achieve trustworthy information sharing among vehicles [8,40,49,50]. For instance, Yi et al. [43] proposed a blockchain-based Public Key Infrastructure for secure verification of vehicular networking devices. Adja et al. [47] introduced DARVAN, which minimizes exposure of critical data while ensuring management and verification of data. Kaur et al. [45] introduced a cross-data center authentication and key exchange scheme for vehicular networks based on blockchain and elliptic curve cryptography (ECC). In the field of financial system security, Youssef et al. [41] proposed a blockchain-based infrastructure for small-scale payments that dynamically adjusts verification levels and detects user misconduct based on historical behavior and real-time data. In the field of building facility security, Halgamuge et al. [39] proposed a new platform architecture for bridge monitoring applications. This architecture implements authenticated data deletion policies to enhance the sustainability of cloud databases. The authors utilized blockchain and smart contracts to ensure the data deletion policies. In addition, researchers have also explored deploying deep learning algorithms on blockchain to jointly serve data verification processes [52]. The above research highlights the impact on the food and agriculture sector, where the design of communication system research methods enhances the security and reliability of data in agricultural decision-making processes by ensuring the verifiability of IoT data streams, which is critical for precision agriculture and food safety monitoring. Additionally, the design of research methods for energy systems, transportation systems, financial systems, and building facility security contributes to the safe sharing of energy resources in a networked environment, the maintenance and authentication of data shared between vehicles, the reduction in transaction verification complexity, and the authentication of data and human behavior. These improvements enhance the energy efficiency of agricultural mechanization and food processing, increase the efficiency and safety of agricultural product logistics, ensure the security of agricultural financial services, and safeguard food processing and storage facilities, thereby positively impacting the overall operations and efficiency of the food and agriculture sector.

Researchers employ BT’s decentralized architecture and programmable smart contracts to achieve highly automated and trustworthy data validation in the OTCI field. This approach is aimed at safeguarding CI against network attacks.

#### 4.1.2. Security Access Control

In the OTCI field, there is a lack of data, operational, and personnel permission management, as well as access authorization control. Strengthening the security access control mechanisms in OTCI can play a significant role in defense against network attacks before they occur. Researchers have explored the use of BT for distributed access control in OTCI to enhance the reliability and security of CI. The research focus is distributed across various fields, including transportation systems, healthcare systems, communication systems, energy systems, financial systems, industrial control systems, building facility security, and food and agriculture systems, as shown in Table 7.

In the field of transportation system security, research primarily focuses on Vehicular Ad Hoc Networks (VANETs). VANETs allow real-time data exchange between vehicles, roadside units, parking lots, and city infrastructure. Therefore, strengthening the security access control of VANETs can enhance the network security defenses of CI in the transportation system [67,72]. For example, Sharma et al. [71] proposed the integration of BT into dedicated vehicular networks, allowing vehicles to use a distributed access control system for sharing. This ensures that shared network resources have a higher level of trust, reliability, and security. Awais et al. [66] introduced a secure distributed messaging framework to enhance security and reduce traffic. In the field of healthcare system security, Dubovitskaya et al. [70] developed patient access control strategies specifically aimed at enhancing the privacy and high sensitivity of electronic health records (EHRs). They proposed a blockchain-based permission system for EHR data sharing and integration. In the field of communication system security, researchers have explored the use of BT in various areas of IoT communication, including Software-Defined Networking (SDN), Wireless Sensor Networks (WSNs), Border Gateway Protocol (BGP), and 5G. They have investigated the implementation of cross-domain identity authorization for individuals [60], internet number resource permission and trust management [63,68], and lightweight stakeholder identity authentication authorization [69], all aimed at enhancing information system security. In communication systems, the sheer number of heterogeneous IoT devices poses a significant challenge. Research based on blockchain’s decentralized architecture, certificate systems, and programmable smart contracts can achieve secure access and control among IoT devices [62,64]. Furthermore, in the context of mobile data access, Xenakis et al. [65] proposed a new blockchain-supported mobile data access model in conjunction with 5G to ensure secure user access. In the field of energy system security, research primarily focuses on battery energy storage systems and smart grid applications. For example, Mhaisen et al. [61] introduced a new control method for battery energy storage systems based on distributed smart contracts to achieve their collaborative and secure operation. Suciu et al. [55] introduced a layered architecture composed of different permission entities for managing physical entities’ access to various resources in the network infrastructure. In the field of financial system security, Ivanov et al. [58] designed VolgaPay, an offline payment terminal, and VolgaGuard, an offline resource access control terminal, based on BT for self-service terminals (SSTs). This design aims to enhance the resilience of self-service terminal systems against network attacks. In the field of industrial control system security, Halgamuge et al. [57] established a comprehensive latency model for distributed CI access control using blockchain. It employs a decentralized Security Access Administrator (SAA) instead of a centralized Certificate Authority (CA) to provide distributed access control. In the field of building facilities security, two key issues when using BT for modular construction are the collection and real-time updating of data and the management of authorized data access. Kong et al. [59] proposed a permissioned blockchain architecture with an IoT oracle, utilizing blockchain networks to provide authentication and ensure authorized access to quality inspections. In the field of food and agricultural system security, Gaba et al. [56] highlighted the need to protect personal data related to agricultural land records in the agricultural system security domain. The aim is to prevent unauthorized access and eliminate corruption in land transactions. The author proposes a solution based on distributed ledger technology. In the impact of the above research on the food and agriculture sector, ensuring secure communication within vehicle networks has improved the efficiency of agricultural product logistics and reduced food waste. Meanwhile, the secure sharing of electronic health records helps safeguard the health of agricultural workers and enhances productivity. The management of precision agriculture data, the application of blockchain technology in energy and quality control, and the cybersecurity protection of financial services collectively strengthen the safety and efficiency of the food and agriculture sector.

In the realm of OTCI, BT offers decentralized solutions for identity authentication, data verification, and secure access control. When combined with technologies such as machine learning, 5G, neural networks, and others, it collectively enhances the security of data sharing. This, in turn, strengthens the boundary defense capabilities of OTCI, serving as a preventive measure before a CI comes under attack.

### 4.2. During-the-Attack of OTCI

#### 4.2.1. Attack Detection and Perception

When CI is subjected to network attacks, detecting and perceiving attack viruses, methods, and pathways can provide robust defense support for CI after the attack. Scholars have explored the combined application of programmable smart contracts, blockchain signature systems, consensus mechanisms, and neural networks and machine learning algorithms to study OTCI attack detection and perception. The research is primarily focused on fields such as industrial control systems, energy systems, transportation systems, communication systems, supply chain management, financial systems, and BT, as shown in Table 8.

In the field of ICS security, Ragab et al. [73] have designed a new blockchain with deep learning-based cyberattack detection (BDLE-CAD) for CI and ICS. They have proposed a blockchain-supported integrity check scheme (BEICS) to defend against error route attacks. Demertzis et al. [78] utilized smart contracts to achieve bilateral traffic control and detect anomalies based on a Deep Autoencoder Neural Network (DANN). Kumar et al. [91] introduced a novel integrated framework of blockchain and deep learning to safeguard sensitive information and identify network threats based on network traffic analysis. In the field of energy system security, research is primarily focused on smart grids. The modernization of the grid involves increased use of “smart” energy devices that can automate, digitize, network, and integrate the physical energy supply chain within the grid. Therefore, in the environment of the energy IoT, relevant infrastructure is highly susceptible to network attacks. Mylrea et al. [77] explored how BT can enhance the compliance of the North American Electric Reliability Corporation (NERC) CIP by using immutable cryptographic signatures and distributed ledgers, thereby increasing the security of the bulk power system supply chain. Sai et al. [74] introduced the Hybrid AI Blockchain-Supported Protection Framework (HABPF). It utilizes Recurrent Neural Networks (RNNs) and a Convolutional Neural Network (CNN) based on LeNet5 to protect the communication infrastructure of the smart grid. Furthermore, researchers have explored the use of custom consensus mechanisms, traceability, and anonymity to provide a trusted environment and necessary conditions for attack detection, thus aiding in attack perception [79,85,87]. In the field of transportation system security, researchers have explored the use of novel distributed detection and response methods to perform network situational awareness and attack detection in transportation systems. Graf et al. [76] utilized smart contract technology to provide an automated and trusted system for event management throughout its lifecycle. This system allows for the automatic acquisition, classification, use, archiving, and disposal of events. Finogeev et al. [81] employed BT to validate network nodes and store sensor data in a distributed ledger. It introduces a network data packet clustering method based on fuzzy neural networks, which can be used to detect data packets that may contain malicious content. Vargas et al. [80] established a comprehensive security mechanism for device networks in the IoT. It focuses on identifying threats while considering the computational capabilities suitable for industrial IoT. In the field of communication system security, scholars have conducted research on leveraging BT in conjunction with fog computing, deep learning, signature mechanisms, and decentralized frameworks. These technologies are used for monitoring devices and smart contracts [92,93,94,95], detecting Distributed Denial of Service (DDoS) attacks [89], and identifying malicious activities such as intrusions [90] and zombie networks [75]. Furthermore, in practical applications, Bernieri et al. [88] introduced ALISI, a lightweight identification system designed specifically for IoT and IIoT systems. In the field of financial systems, Zhang et al. [84] have proposed a threshold estimation framework based on blockchain and deep learning for exchanges. This framework can be utilized to estimate the optimal threshold for hot wallets within complex and dynamic exchange environments, assisting in attack detection. With the expansion of BT application scenarios and its ongoing development, blockchain is gradually evolving into a new form of CI. In the research related to blockchain’s own attack perception and detection, Eisenbarth et al. [82] have proposed an architecture to detect suspicious nodes and revoke them in order to mitigate future Sybil attacks. Zhang et al. [83] introduced SVScanner, which utilizes heterogeneous feature patterns to detect vulnerabilities in smart contracts within the blockchain. Alangot et al. [86,96] proposed two methods for Bitcoin clients to detect if eclipse attacks are being conducted against them. In the impact of the above research on the food and agriculture sector, the anomaly detection technology in industrial control systems protects critical data in agricultural automation, ensuring the continuity and safety of food production. At the same time, attack detection in energy and transportation systems enhances the stability of agricultural energy supply and logistics, reducing food waste. Security measures in communication and finance sectors improve the transparency of the supply chain and the safety of funds, while the application of blockchain technology enhances the efficiency and transparency of the agricultural supply chain.

The above-mentioned research has explored the role of BT in attack perception and protection in OTCI networks. Blockchain can provide a trustworthy environment for detection, and through highly automated smart contracts, signature mechanisms, and consensus mechanisms, it can introduce innovative distributed detection methods into traditional approaches. Combining BT with traditional attack detection algorithms yields significant advantages in safeguarding OTCI networks. However, BT, as a CI, also faces certain network security risks. Currently, there is a lack of research on how to integrate BT’s own threat perception and detection with OTCI.

#### 4.2.2. Data Security and Protection

With the interconnection of CI control systems, data interactions occur over the internet using protocols such as IP/TCP. Compared to traditional infrastructure control and data exchange, the speed and breadth of data sharing have significantly improved. However, this increased data flow has also led to a rise in network attacks due to the decentralized and immutable nature of BT, where all nodes collectively maintain the security of data on the chain. For example, in a blockchain network, each node participates in the consensus of the network, and every request undergoes a voting and validation process by all nodes on the chain. Researchers have explored the use of blockchain for data security and protection in CI. The research primarily focuses on two aspects: data transit protection and data storage protection.

i. Data Transit Protection

To protect the data transit process in the OTCI field, researchers have primarily explored the use of blockchain’s distributed framework, smart contracts, consensus mechanisms, encryption methods, and more to safeguard data transit processes. The research is primarily focused on the fields of communication systems, financial systems, energy systems, healthcare systems, electronic voting, transportation systems, cloud computing, and BT itself, as shown in Table 9.

In the field of communication system security, the research includes using BT for data integrity checks [10], data flow monitoring [111], and data traceability in communication systems [116]. Mena et al. [99] incorporated real-time transaction mechanisms into smart contracts to ensure that negotiated contract commitments are fulfilled. Moges et al. [103] stored changes in network states on an immutable ledger to provide reliable traceability. Wang et al. [105] presented a secure spectrum optimization solution for satellite IoT, introducing BT to deter malicious users from participating in spectrum sharing. Sunny et al. [108] have developed a lightweight framework based on blockchain, utilizing its layered nature to protect CI. In the field of financial system security, Zhang et al. [97] leveraged a combination of public key encryption schemes, digital signature schemes, and smart contracts to facilitate four tasks in intelligent auctions: data submission, data requests, auctions and queries, and payment and delivery. In the field of energy system security, researchers explore the use of encryption algorithms (blind signature [98]) and distributed architectures [109] to achieve secure energy transactions and remote monitoring. In the field of healthcare system security, the research combines blockchain with post-quantum encryption algorithms [100], digital twin technology [115], neural network algorithms [106], and more to perform real-time data processing, avoid single points of failure, and ensure the secure sharing of sensitive medical data (such as medical records). This approach aims to enable trusted data sharing and precision healthcare. In the field of electronic voting system security, researchers primarily investigate the use of blockchain to ensure the integrity of voting data, thus safeguarding the security of data flow [101]. In the field of transportation system security, dealing with a mobile network composed of vehicles, roadside units, and other infrastructure, researchers primarily explore the use of blockchain along with modular architecture [112], certificate authentication mechanisms [102], and smart contracts [107] to ensure trusted data circulation while securing data interactions between vehicles. Comprehensive data protection is applied to data circulation in scenarios such as road congestion control [104], carbon emissions trading, and others, with the aim of improving road safety and traffic control. In the field of cloud computing, Khan et al. [110] presented a blockchain-based distributed infrastructure that leverages fundamental blockchain attributes to achieve immutable and trustworthy service level monitoring within cloud services. In the field of food and agriculture safety systems, scholars have primarily focused on providing a transparent, decentralized blockchain-tracking solution for agricultural production. They proposed a rice supply chain refinement supervision model MBRRSM (Multi Blockchain Rice Refinement Supervision Model) based on multi-layer blockchain from the information level, which is used to increase transparency and reduce the circulation of problematic rice [114]. In the domain of BT itself, to ensure the confidentiality of sensitive data, scholars have employed encryption methods to secure data collection, data search, and data processing [113,117,118]. In the impact of the above research on the food and agriculture sector, enhanced communication network security protects the transmission of food and agricultural data, ensuring the security of the supply chain. BT improves the transparency of energy management, reduces costs, while also improving the quality of healthcare services, safeguarding agricultural product logistics efficiency, enhancing the reliability of cloud services, and increasing the overall trustworthiness of the agricultural supply chain.

The above-mentioned research leverages the decentralization, tamper-proof, and anonymity features of blockchain to monitor and protect the integrity of data throughout its lifecycle. Furthermore, through the customization of smart contracts and the design of encryption mechanisms, human involvement is minimized, facilitating end-to-end on-chain operations for data transmission, processing, and decision-making. In the OTCI security domain, ensuring the security of data flow has significant advantages compared to traditional centralized data flow protection methods in terms of security, trustworthiness, and cost control. However, existing research only guarantees the trustworthy flow of data on the blockchain, and there are still risks in the data flow from collection to storage.

ii. Data Storage Protection

In the field of CI, data storage security forms the foundation of network defense. BT can provide a trusted environment for data storage in CI. Researchers have primarily focused on the use of BT in the field of network security for CI, with a particular emphasis on industrial control systems, communication systems, and cloud storage, as indicated in Table 10.

In the field of industrial control system security, researchers primarily utilize BT to protect data during the training and testing phases of machine learning models [119] and maintain data throughout the data storage of the entire industrial control system [123]. This approach enables defense against four types of attacks: random label flipping, target label flipping, fast gradient sign method, and Jacobian saliency map attacks. In the field of communication system security, researchers have created a distributed environment based on BT to prevent data forgery or loss during the communication phase of information–physical systems [120,125]. Furthermore, Otoum et al. [121] designed a blockchain-federated learning model. This model decentralizes the learning process and utilizes blockchain to securely protect the transmission of intermediate parameters, ensuring the privacy and security of critical IoT infrastructure systems. In the field of cloud storage, researchers have proposed a security data source framework for cloud-centric IoT networks. They have achieved this by leveraging blockchain smart contracts in conjunction with the immutability, determinism, and public nature of traditional cloud infrastructure. This framework is designed for securely recording data operations occurring in cloud environments. In the impact of the above research on the food and agriculture sector, the design of industrial control system research methods protects the accuracy and reliability of production data, which is critical for the food and agriculture sectors. The design of communication systems prevents data falsification or loss, enhancing the security of food traceability and supply chain management. The design of cloud storage frameworks improves the efficiency and security of cloud-based processing of agricultural data, including crop monitoring and market data analysis.

Scholars leverage BT to provide a trustworthy data storage environment. Through smart contracts, data are comprehensively recorded, and their security is maintained through collaborative efforts from network nodes. This research offers higher security compared to traditional centralized storage methods. However, it is worth noting that scholars have not extensively delved into the study of storage costs. BT poses challenges in terms of storage performance and costs when applied to large-scale storage processes.

### 4.3. Post-Attack of OTCI

After a cyberattack on CI and the removal of the attack’s malware, the recovery, assessment, and attribution of relevant data play a vital role in rapidly restoring the CI to normalcy and providing targeted protection against future network attacks. Scholars mainly explore the use of BT in the recovery, assessment, and attribution of CI after cyberattacks.

#### 4.3.1. Data Backup and Recovery

To the data redundancy mechanism on the BT, where all nodes collectively maintain and store the entire data or relevant data hashes, BT ensures data integrity and prevents data loss. It also enables rapid, secure, and trustworthy data recovery. Scholars primarily focus on the fields of the IoT, healthcare systems, and ICS, as shown in Table 11.

In the field of IoT security, Alfandi et al. [127] constructed a blockchain-based architecture for the IoT to provide distributed storage functionality. Through the Byzantine Fault Tolerance (BFT) mechanism, CI can continue functioning even when one or more of its components fail. In the field of healthcare system security, to ensure real-time tracking and monitoring of patient health, scholars have eliminated single points of failure by distributing data and interaction records on a blockchain network. This ensures the trustworthy recovery of the electronic healthcare system [128]. In the field of industrial control systems, network attacks on log devices can jeopardize any forensic analysis, whether it is used for maintenance or discovering traces of attacks. Researchers use blockchain to protect factory operational data stored in historical databases and data exchanges in the network. They support efficient replication mechanisms to recover data after attacks [126,129]. In the impact of the above research on the food and agriculture sector, the blockchain architecture for the internet of things enhances the security of agricultural data and the traceability of the food supply chain. At the same time, the blockchain distribution of medical data improves the security of agricultural workers’ health information and strengthens trust in healthcare services for agricultural communities. The data recovery mechanism of industrial control systems ensures the rapid recovery of agricultural production after a cyberattack, reducing the impact on the food supply chain.

The above-mentioned studies primarily leverage BT for comprehensive data tracking and distributed redundant storage to achieve data backup and recovery. This facilitates rapid, secure, and cost-effective data recovery in the event of network attacks on OTCI. However, in the CI sector, not only is the rapid recovery of data after an attack essential, but also enhancing the resilience of CI operations when faced with network attacks is equally important.

#### 4.3.2. Attack Assessment and Accountability

In the OTCI domain, assessing attacks and attributing attack incidents play a significant role in implementing effective network protection measures for designated CI. BT can indeed provide a means to trace and track data operations. Research primarily focuses on the field of communication systems, where accountability is achieved through mechanisms such as signature schemes [133], decentralized architectures [131], and incentive structures. These approaches promote automatic assessment, encourage user participation in audits, and penalize unintentional or malicious activities [134]. For example, Faisal et al. [130] introduced the BEAT (Blockchain-Enabled Accountability and Transparency) infrastructure sharing framework, which enables device-level accountability. Suhail et al. [132] envisioned a blockchain-based digital twin framework, serving as a trusted twin for protecting Trusted Things and Systems in Cyber–Physical Systems (TTS-CPSs). It can track the responsible entities for adding or updating security and safety rules and ensure the trustworthiness of data sources through ICM.

The mentioned research in CI network security indeed provides higher security and automation compared to traditional centralized management models. However, the existing research is primarily focused on attack assessment and accountability. The future research direction may involve the automatic learning of attacks and the deployment of subsequent defense mechanisms.

## 5. Design of a General Framework for the Blockchain of Operational Technology Security in the Critical Infrastructure

**Figure 9:**
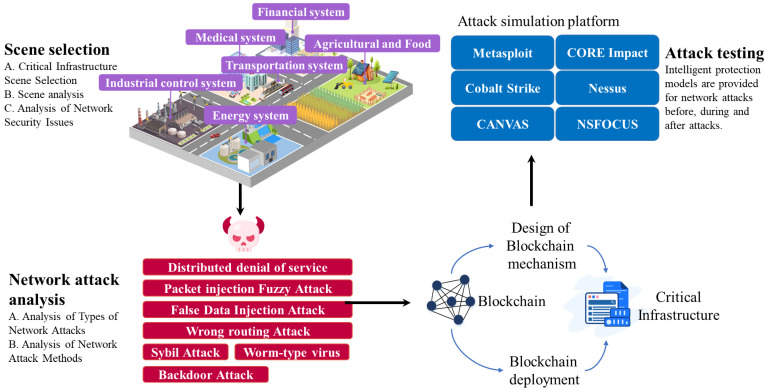
Schematic diagram of the general framework of OTCI research based on BT.

Based on bibliometric analysis and content analysis of CI network security in the OT field, a general framework for blockchain security research in OTCI is proposed in this article, as shown in Figure 9. The general framework is divided into four steps: scenario selection, network attack analysis, blockchain-based network defense model design and deployment, and attack test analysis.

CI areas are divided into 16 major areas by the U.S. Department of Homeland Security, including environmental system security, government facility security, education system security, scientific research system security, industrial control system security, building facility security, digital media system security, social media, and online community security. There are certain differences in data flow methods, infrastructure, stakeholders, life cycles, etc., in each field. Therefore, the first step is to analyze the application field, including traditional network protection methods, scenario characteristics, and related network background requirements.

Secondly, upon confirming the background, our first task is to analyze the types of network attacks suffered in this field, as illustrated in Table 12. Subsequently, the methods employed for each attack are examined, and their distinctive characteristics and commonly used defense measures are identified. It is crucial to pinpoint the occurrence locations of network attacks in CI, which typically encompass external networks, internal networks, various stages of the supply chain, internal logical programs of instrumentation devices, network communications, and data monitoring, among others.

Third, after analyzing the above attacks, automation, decentralization, low score, high efficiency, immutability, and a wide range of blockchain smart contracts are used to solve or optimize problems that cannot be solved by traditional models or technologies in the field of CI network security. The following intelligent protection models are presented, corresponding to OTCI before, during, and after network attacks, as shown in Table 13. After the model design, in order to realize the design function of the model, the related internal mechanisms of the BT are designed, including the customized design of the smart contract, the optimization of the consensus mechanism, the improvement of the storage mechanism, and the design of the encryption algorithm. Then, the development platform, contract development language, deployment mode, etc., are selected. Common development platforms include Ethereum, Hyperledger, Chang’an Chain, Corda, Polkadot, Ergo, etc. Contract development languages include C++, Golang, Java, Solidity, Python, JavaScript, etc. Deployment modes include public chain deployment, private chain deployment, alliance chain deployment, and hybrid chain deployment. Finally, the actual development and deployment are carried out.

Fourth, testing and simulation of network attacks are conducted. Through testing and simulation, the robustness, performance, and weaknesses of the blockchain-based intelligent protection model are tested and fed back to the model design step to optimize the model. Common network attack simulation platforms include Metasploit, CORE Impact, Cobalt Strike, Nessus, CANVAS, etc. At last, the actual scenario is implemented to enhance the network defense capability of CI.

A general framework for OTCI network security research based on BT has been designed, reflecting the steps and methods of BT research in the industry. This framework provides support for subsequent BT research in the field of OTCI network security.

## 6. Discussion

**Figure 10:**
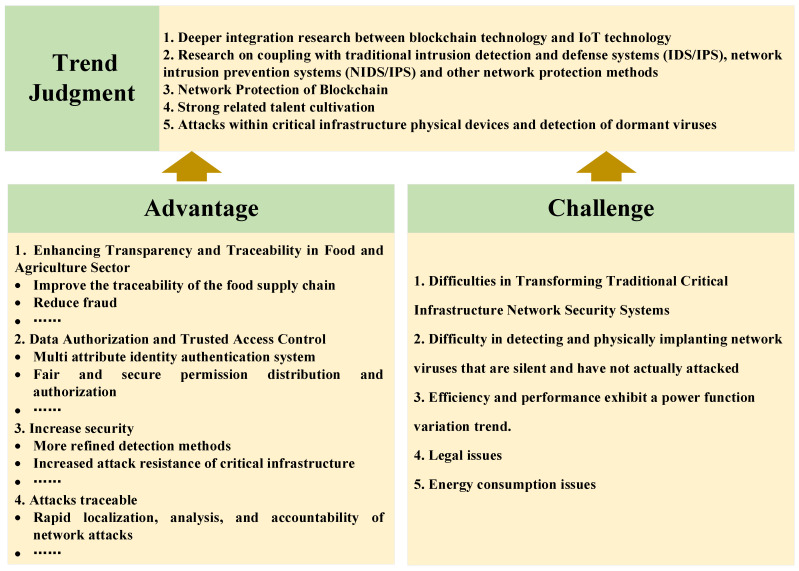
The advantages, challenges, and trends of OTCI network security based on BT.

As the world is moving toward the “Internet of everything”, the networking of OTCI has increased the difficulty of network security, and the risk of cyberattacks on the software layer, hardware layer, communication layer, and application layer of OTCI is increasing. OTCI needs to introduce new technologies to improve network protection capabilities in new environments and contexts. This study presents a comprehensive review of emerging BT research in the field of OTCI cyber security through bibliometric analysis and content analysis. The following section discusses the benefits, challenges, and future research trends for the application of BT in the field of OTCI network security, as illustrated in Figure 10.

### 6.1. Advantage Analysis

Traditional defenses for CI networks include firewalls, intrusion detection and prevention systems, virtual private networks, intrusion prevention systems, antivirus software, access control lists, and security information and event management. However, these measures are plagued by single points of failure, limited real-time capabilities, complex management, inadequacy against advanced threats, lack of transparency, and reliance on signatures and rules. To enhance network security, research and adoption of emerging technologies like blockchain are advocated to address these shortcomings. Blockchain provides decentralized, transparent, and tamper-resistant security mechanisms to counter the growing complexities of network threats. Based on content analysis, six topics for the application of BT in the OTCI field were identified. In previous research, scholars have conducted certain research in the directions of identity authentication and data verification, secure access control, attack detection and perception, data security and protection, data backup and recovery, and attack assessment and attribution. It can be seen from the analysis that the research focuses on the CI network security technology research.

Without a doubt, BT can bring revolutionary changes to traditional OTCI network protection, especially in the food and agriculture sector. Due to its support for secure and immutable records, blockchain can improve the transparency, traceability, and efficiency of the food industry supply chain. This provides beneficial support for reducing food fraud, improving food safety, and adding new logistics channels for agriculture and food distribution. The application of BT to the OTCI field is considered capable of providing full network protection from the data layer to the physical layer, offering the following advantages.

#### 6.1.1. Enhancing Transparency and Traceability in Food and Agriculture Sector

BT offers transformative advantages for enhancing the transparency and traceability of Operational Technology in food and agriculture CI. By leveraging immutable records and decentralized ledgers, blockchain ensures that every step of the supply chain—from origin to processing and distribution—is securely tracked. This minimizes food fraud and improves food safety by allowing rapid identification of contamination sources. For instance, blockchain-based systems can automate recall processes, reducing response times and improving the accuracy of safety interventions. Furthermore, blockchain-powered smart contracts optimize logistics and inventory management in agricultural operations, streamlining food production and distribution while reducing waste.

#### 6.1.2. Data Validation and Trusted Access Control

The use of BT’s decentralized architecture, anonymity, signature mechanism, consensus mechanism, and customizable, programmable smart contracts can provide trusted identity management for OTCI and increase the credibility and efficiency of OTCI supervision. BT can provide a trusted data environment for CI through the irreversibility of hash signatures. It can prevent the tampering of the identity information of operators, operating equipment, etc. In addition, the data on the BT are jointly maintained by all nodes, and the data are left with marks throughout the process, reducing the risk of data loss and damage. And it can provide multi-source and comprehensive data sources for identity authentication and carrying out multi-attribute verification. Second, smart contracts can operate credibly according to preset functions and automate data analysis and verification. This method reduces personnel participation and operating costs, including time, labor, equipment, materials, etc. At the same time, the credibility of the process is improved. In terms of trusted control, the use of BT can bring fair and secure permission distribution to CI. And it can automatically judge the behavior of personnel and equipment through contracts and then automate the control of permissions to achieve trusted data access.

#### 6.1.3. Increase Security

In the OTCI field, the use of BT provides a higher level of security than traditional means of network protection. In attack detection and perception, first, the consensus mechanism of the BT is designed so that every operation and request requires full-chain node authentication. Therefore, it can provide more refined detection means for attack detection of operation requests. Second, BT provides a trusted execution environment for relevant detection algorithms, and the contract-based detection algorithm is more credible. Third, BT’s data storage model provides a trusted basis for the detection and perception of attacks. In terms of the trusted flow and storage of data, BT adopts asymmetric encryption algorithms, which can greatly increase the flow of data and communication security. This is because each request requires consensus voting from all nodes in the chain. In addition, the distributed, full-node storage mode makes the attackers carry out a comprehensive attack on the data of more than 51% of the nodes of the whole chain before the attack can be successful, increasing the anti-attack of CI.

#### 6.1.4. Attack Traceability

After CI is attacked, the traceability of the BT can be used to locate the location of the attack, which can provide credible evidence for the analysis of the attack. The storage mode of the whole trace can realize the reliable responsibility of the attack and reduce the cost of the responsibility.

### 6.2. Challenge Analysis

In the field of OTCI, malicious cyber activities have evolved from deliberate destruction to espionage and intellectual property theft, destructive attacks on CI, and ransomware attacks. The use of BT provides a more secure, efficient, and diverse means of attack protection throughout the entire lifecycle than traditional network security protection. However, BT also brings significant challenges that must be addressed to ensure its wider adoption and sustainability. Especially in the CI sector of the food and agriculture sector, adopting BT will also result in significant electricity consumption. This is due to the highly dependent level of computing power based on the proof-of-work mechanism, which leads to the blockchain network consuming a large amount of electricity. This type of electricity consumption not only brings some carbon emissions but also indirectly affects the entire food cycle by increasing competition for resources such as land and water (now converted into energy production). In this situation, the energy structure required for mining activities may conflict with agricultural demand, thereby exacerbating food insecurity in areas already facing resource scarcity pressures.

Blockchain’s energy-intensive nature exacerbates resource competition, particularly in regions reliant on energy-intensive proof-of-work (PoW) mechanisms. The high electricity consumption associated with blockchain operations not only leads to increased carbon emissions but also indirectly impacts critical resources like land and water, which are often diverted to energy production. This competition can significantly affect agricultural productivity and food security in resource-constrained regions.

Additionally, the transformation of traditional CI network security systems poses technical challenges, including the complexity of integrating blockchain with existing OT, training personnel, updating equipment, and building trust in decentralized systems. Secondly, while blockchain’s consensus mechanisms, encryption algorithms, and signature systems are designed for effective real-time detection during network attacks, they are less effective in identifying dormant threats, such as silent network viruses or physically implanted malware. A comprehensive approach combining blockchain with traditional detection methods is necessary to enhance its effectiveness.

Furthermore, performance and efficiency issues remain significant obstacles. As data volume, node numbers, and operational demands increase, blockchain systems may face concurrency and operational delays, limiting their scalability in OTCI environments. Legal and regulatory challenges also hinder blockchain adoption, as compliance with existing regulations and the development of legally sound smart contracts remain complex tasks.

Finally, in the context of sustainability, the resource-intensive nature of blockchain operations must be addressed. Alternative consensus mechanisms, such as proof-of-stake (PoS), directed acyclic graphs (DAGs), and other low-energy architectures, offer promising solutions. Simultaneously, integrating renewable energy sources into blockchain operations can alleviate resource pressures while ensuring environmental sustainability.

### 6.3. Trend Analysis

Considering the advanced persistent threats faced by OTCI, trend development research and evaluation have been conducted on the application and technical aspects of BT. Key areas of focus include the integration of BT with IoT technology, deep learning, and machine learning algorithms to enhance functionality; the coupling of BT with traditional network protection mechanisms such as firewalls, intrusion detection and prevention systems (IDS/IPS), network intrusion prevention systems (NIDS/IPS), and packet filters; and the advancement of network security research on BT as a new generation of CI, including anti-quantum encryption algorithms, lightweight blockchain, directed acyclic graphs, and more efficient and secure architectural systems [17]. Additionally, challenges such as the insufficient talent pool for blockchain smart contract technology in OTCI are highlighted, necessitating the establishment of specialized training mechanisms, dedicated BT management positions, and improved contract management frameworks. Research on BT-based detection of dormant network viruses within physical devices in OTCI environments is also emphasized. As the results of this study are dynamic and subject to change, the inclusion of new publications, adhering to the September 2023 deadline, is recommended, and periodic updates of this review in future years are suggested.

## 7. Conclusions

As food and agriculture infrastructure becomes increasingly interconnected and digitalized, blockchain technology (BT) emerges as a transformative tool to address the critical challenges of network security, traceability, and resilience in this domain. This study conducts a comprehensive review by employing bibliometric analysis and content analysis of previous research articles. It aims to reveal the current state of research on BT in OTCI, the challenges it faces, and future development trends. The bibliometric analysis concludes that the main research areas are computer science, engineering, medicine, and environmental science. Regarding development, since 2018, there has been a significant increase in publications on this topic, which has continued in a steady manner until the end of the analysis period. This trend provides a clear understanding of the importance of BT in OTCI research and allows for predictions of growth in the coming years. The leading countries and institutions in this field are the United States, China, and India. Among the most influential authors, Otoum Safa has published the most papers and has the highest work efficiency. Asuquo Philip, Cao Yue, Cruickshank Haitham, Lei Ao, Ogah Chibueze P. Anyigor, and Sun Zhili have the highest citation counts (all with 384 citations) and the highest recognition for their work. On the other hand, the various bibliometric tools used enable the establishment of key research paths, providing useful information for researchers interested in BT in OTCI. The results of the bibliometric analysis contribute to the development of new research, facilitate international collaboration, and provide researchers with the latest opportunities and research gaps in the BT field within OTCI. As food and agricultural infrastructure is a key component of OTCI, the bibliometric analysis helps promote the secure development of food and agricultural infrastructure. In terms of content analysis, six key topics have been outlined, including identity authentication and data validation, secure access control, attack detection and perception, data security and protection, data backup and recovery, and attack assessment and attribution. These findings provide a valuable foundation for exploring the specific applications of BT in food and agriculture systems, a vital subset of CI. Moreover, a general framework for research on BT in OTCI was designed. This study serves to identify the influential areas and publication channels of BT research in OTCI. It provides valuable insights for scholars to gain a comprehensive understanding of recent hot topics and technological trends. Finally, the proposed general framework serves as a guide for integrating BT into food and agriculture operations, from ensuring trusted traceability across supply chains to securing OT in smart farming and food processing. This review can foster the advancement of BT in OTCI, with a specific emphasis on strengthening the security, resilience, and efficiency of networks within food and agriculture systems.

This review has contributed, to some extent, to the development of BT in OTCI, offering valuable insights. However, this study also comes with certain limitations:

- The research on BT in OTCI has been reviewed; however, there is a limitation in conducting an in-depth analysis of BT research from a computer science perspective;
- Research on BT in the OTCI is a dynamically evolving field. It is important to note that this study focuses on literature up until September 2023, which means it may not cover the most recent developments in this area;
- This review primarily provides an overview of BT research within OTCI. The defined six topics have a limited scope, and there is a possibility that some relevant and significant topics may not have been included in the analysis.

## Figures and Tables

**Table 1:** Analysis table of review articles.

Journal	Year	Field	Analytical Method	Censorship Content
[11]	2020	Internet of things	Content analysis	IoT/IIoT CI security review in Industry 4.0
[12]	2019	Healthcare, Cyber–Physical Systems	Content analysis	An introduction to critical national infrastructure cybersecurity
[13]	2022	Internet of things	Content analysis	The current state of the art of different IoT architectures, as well as current technologies, applications, challenges, IoT protocols, and opportunities
[14]	2021	Energy system	Content analysis	Architecture and functionality of IoT-enabled smart energy grid systems
[15]	2021	Internet of things	Content analysis	Security and privacy challenges related to IoT

**Table 2:** Definition of CI sectors [11].

Sector	China	U.S.	Germany	Russia
Chemical Sector		√		
Commercial Facilities Sector		√		
Communications Sector	√	√	√	√
Critical Manufacturing Sector	√	√		
Dams Sector	√	√	√	
Defense Industrial Base Sector		√		√
Emergency Services Sector		√		√
Energy Sector	√	√	√	
Financial Services Sector	√	√	√	
Food and Agriculture Sector		√	√	
Government Facilities Sector	√	√	√	√
Healthcare and Public Health Sector	√	√	√	
Information Technology Sector		√		
Nuclear Reactors		√		
Materials and Waste Sector		√		
Transportation Systems Sector	√	√	√	√
Water and Wastewater Sector	√	√	√	
Technology/Research Sector	√			√
Education Sector	√			
Social Security Sector	√			
Important Internet Applications Sector	√			
Judicial Sector				√
Media and Culture Sector			√	

**Table 3:** Country distribution table.

No.	Country	Number of Documents	Number of References	Total Citations
1	USA	30	839	347
2	China	23	946	236
3	India	21	954	200
4	Saudi Arabia	12	538	31
5	Pakistan	11	534	108
6	United Kingdom	9	467	507
7	Canada	9	199	103
8	Australia	8	263	92
9	United Arab Emirates	7	290	87
10	Singapore	6	171	97

**Table 4:** Analysis of research topics.

Pre-Attack Stage of OTCI	Identity Authentication and Data Validation	Identity Authentication	[9,19,20,21,22,23,24,25,26,27,28,29,30,31,32,33,34,35,36,37]
		Data Validation	[8,38,39,40,41,42,43,44,45,46,47,48,49,50,51,52,53,54]
	Security Access Control		[55,56,57,58,59,60,61,62,63,64,65,66,67,68,69,70,71,72]
During-the-Attack Stageof OTCI	Attack Detection and Perception		[73,74,75,76,77,78,79,80,81,82,83,84,85,86,87,88,89,90,91,92,93,94,95,96]
	Data Security and Protection	Data Transit Protection	[10,97,98,99,100,101,102,103,104,105,106,107,108,109,110,111,112,113,114,115,116,117,118]
		Data Storage Protection	[119,120,121,122,123,124,125]
Post-Attack Stage of OTCI	Data Backup and Recovery		[126,127,128,129]
	Attack Assessment and Attribution		[130,131,132,133,134]

**Table 5:** OTCI identity authentication.

Literature	Field		Types of Attacks	Effect	Impact on the Food and Agriculture Sector
[19,29,31,32,33,36,37]	Communication Systems	Internet of Things	Replay Attacks, Man-in-the-Middle Attacks, DNS Cache Poisoning, Distributed Denial of Service (DDoS) Attacks	Reducing authentication costs, enhancing authentication efficiency, and bolstering security	Leading to supply chain disruptions, affecting food supply and prices, as well as consumer trust in food brands
[34]		Internet			
[20,23,24]	Energy Systems		Data Injection	Ensuring system resilience, reducing penetration rates, and enhancing the security of smart grid operational management	Affecting the stability of energy systems, thereby impacting the operation of food processing and agricultural machinery
[9,25,26,28,30]	Transportation Systems		Physical, Side-Channel, and Clone Attacks	Reducing authentication overhead and improving authentication efficiency	Impacting the logistics and distribution of food and agricultural products, leading to supply chain delays or disruptions
[22,27]	Industrial Control Systems		Denial of Service Attacks, Data and Identity Deception, and Data Poisoning	By ensuring the integrity and authenticating the identities of underlying sensor data, the reliability of ICS/SCADA ecosystems is enhanced	Resulting in the tampering of sensor data in industrial control systems, affecting food safety and production efficiency
[21]	Social Media and Online Communities		Identity Spoofing, Password Guessing and Cracking, and so on	Ensure the trustworthiness of user identities on proprietary social media platforms	Attacks on social media affect consumer trust in food brands and influence purchasing decisions
[35]	Healthcare System		Attacks, Side-Channel Attacks, Impersonation Threats, Man-in-the-Middle Attacks, and others	Perform mutual authentication between biometric sensor units and hospital medical servers	Attacks on healthcare systems indirectly impact the food and agriculture sector, as they may affect the health and safety of food processing workers

**Table 6:** OTCI data validation.

Literature	Field	Research Method	Effect	Impact on the Food and Agriculture Sector
[38,46,48,53,54]	Communication System	Method Design	Provides data auditing to ensure that IoT data streams offer the organization verifiable information, enabling the secure execution of critical decision-making processes	By ensuring IoT data streams provide verifiable information, the security and reliability of data in agricultural decision-making processes are enhanced, contributing to precision agriculture and food safety monitoring
[42,44]	Energy System	System/Framework Development	Secure sharing of energy resources among users and devices in a networked environment	Secure sharing of energy resources in a networked environment can improve the energy efficiency of agricultural mechanization and food processing, reducing costs
[8,40,43,45,47,49,50]	Transportation System	Method Design	Maintains and authenticates shared data to achieve trustworthy information sharing among vehicles	Ensuring the maintenance and authentication of data shared between vehicles enhances the efficiency and safety of agricultural product logistics, minimizing losses during transportation
[41]	Financial System	Method Design	Reduces the complexity of transaction verification, minimizes losses, and prevents various network attacks	Reducing the complexity of transaction verification, minimizing losses, and preventing cyberattacks contribute to the security and efficiency of agricultural financial services, safeguarding the funds of farmers and agricultural enterprises
[39,52]	Building Facility Security	Architecture/Method Design	Authenticates data and human behavior to ensure the security of building facility-related data	Authenticating data and human behavior to ensure the security of building-related data is critical for the safety of food processing and storage facilities, helping to maintain food safety

**Table 7:** OTCI security access control.

Literature	Field	Research Method	Effect	Impact on the Food and Agriculture Sector
[66,67,71,72]	Transportation Systems	Method Design	Facilitates secure communication between users in the connected vehicle network	Ensuring secure communication between users in vehicle networks improves the efficiency and safety of agricultural product logistics, reducing losses during transportation and minimizing food waste
[70]	Healthcare Systems	Case Study	Ensures secure sharing of electronic health records	Securing the sharing of electronic health records helps monitor the health of agricultural workers, thereby enhancing agricultural productivity and food safety
[60,62,63,64,65,68,69]	Communication Systems	Method Design	Achieves personnel and IoT device access management and trusted authorization, ensuring trusted data sharing	Implementing access management and trusted authorization for personnel and IoT devices ensures the trusted sharing of agricultural data, improving the decision-making quality and efficiency of precision agriculture
[55,61]	Energy Systems	Method Design	Implements access control for data and other resources using blockchain and smart contracts	Using blockchain and smart contracts to enforce access control for data and other resources enhances the transparency and efficiency of agricultural energy use, reducing costs
[58]	Financial Systems	Method Design	Replaces permissive API with multi-signature transaction tokens, enhancing network defense resilience in self-service terminal systems	Replacing APIs with multi-signature transaction tokens strengthens the cybersecurity of self-service terminal systems, safeguarding agricultural financial services
[57]	Industrial Control Systems	Method Design	Enables distributed access control through authentication and authorization	Achieving distributed access control through authentication and authorization improves the security and efficiency of agricultural automation systems
[59]	Building Facilities	Method Design	Utilizes a blockchain network to provide authentication functions, ensuring authorized access to channels related to quality inspections	Leveraging blockchain networks to provide authentication functionality ensures authorized access to quality inspection-related channels, enhancing the safety of food processing facilities and product quality
[56]	Food and Agriculture Systems	Case Study	Protects personal data related to agricultural land records and prevents unauthorized access	Protecting personal data related to agricultural land records prevents unauthorized access, ensuring the security of agricultural production data and safeguarding farmers’ rights

**Table 8:** OTCI attack detection and perception.

Literature	Field	Attack Detection/Perception Categories	Effect	Impact on the Food and Agriculture Sector
[73,78,91]	Industrial Control Systems	False Routing Attacks	Detect anomalies in relevant infrastructure operational parameters while ensuring anonymity and confidentiality of industrial information	Detecting abnormal operating parameters in industrial control systems protects the anonymity and confidentiality of industrial information, which is crucial for agricultural automation and food processing, preventing the tampering of critical production data
[74,77,79,85,87]	Energy Systems	False Data Injection Attacks (FDIAs), Distributed Denial of Service (DDoS) Attacks	Detect/perceive attacks involving data, users, and services within smart grids, energy transmission systems, and bulk power systems to enhance detection resilience	Detecting data, user, and service attacks in smart grids, energy transmission systems, and large power systems enhances detection resilience, which is vital for ensuring the energy supply for agriculture and the energy demands of food processing
[76,80,81]	Transportation Systems	Packet Injection (Fuzzing) and Denial of Service (DoS) Attacks	Perform real-time and automated network situational awareness and attack detection in transportation systems	Real-time and automated network situational awareness and attack detection are crucial for ensuring the security and efficiency of agricultural product logistics and distribution systems, reducing transportation disruptions and food waste
[75,88,89,90,92,93,94,95]	Communication Systems	Botnet Attacks, DDoS Attacks, Intrusion Attacks	Create a trusted environment and signature-based detection schemes for effective detection of known attacks	Creating trusted environments and signature-based detection schemes to effectively detect known attacks is essential for protecting agricultural communication networks and food safety communications
[84]	Financial Systems	Theft Attacks	Assist in attack detection through threshold estimation of hot wallets	Assisting attack detection through threshold estimation of hot wallets is crucial for safeguarding agricultural financial services and farmers’ funds
[82,83,86,96]	BT	Sybil Attacks, Eclipse Attacks	Detecting attacks on blockchain nodes, smart contracts, and more	Detecting attacks on blockchain nodes, smart contracts, and other components is critical for protecting agricultural supply chains and blockchain applications related to food safety

**Table 9:** OTCI data transit protection.

Literature	Field	Research Method	Effect	Impact on the Food and Agriculture Sector
[10,99,103,105,108,111,116]	Communication Systems	Method Design	Enhancing network communication security based on optimal resource allocation	Helps ensure the secure transmission of sensitive data in the food and agriculture sectors, safeguarding the security of agricultural supply chains and market information exchange
[97]	Financial Systems	Method Design	Ensuring the security and fairness of trading network infrastructure	Protects the interests of farmers and agricultural businesses in financial transactions
[98,109]	Energy Systems	Method/Scheme Design	Using BT in smart grids to provide integrity and anonymity	Enhances the transparency of energy usage in the food and agriculture sectors, reduces energy costs, and strengthens energy management
[100,106,115]	Healthcare Systems	Case Studies/Method Design	Maintaining the security and privacy of collected healthcare-sensitive data	Helps protect the health information of food and agriculture workers, improving the quality of healthcare services in agricultural communities
[101]	Electronic Voting	Method Design	Protecting the integrity of voting data	Contributes to the fairness and transparency of the voting process in food and agriculture policy decision-making
[102,104,107,112]	Transportation Systems	Method Design	Ensuring integrity, availability, reliable information exchange, and source trustworthiness of vehicle-related data	Improves the logistics efficiency and security of food and agricultural products
[110]	Cloud Computing	Method Design	Enabling accurate execution of service level agreements (SLAs) by all stakeholders through publicly maintained immutable ledgers in a blockchain network, without inaccuracies or delays	Increases the efficiency and reliability of cloud services in the food and agriculture sector
[114]	Food and Agriculture Systems	Scheme Design	Improving security, privacy, transparency, and trust among all stakeholders in agricultural supply chains	Enhances the security, privacy, transparency, and trustworthiness of all stakeholders in the agricultural supply chain
[113,117,118]	BT	Scheme Design	Security of data collection, data retrieval, and data processing	Protects the integrity of agricultural data, contributing to the accuracy of agricultural decision-making

**Table 10:** OTCI data storage protection.

Literature	Field	Research Method	Effect	Impact on the Food and Agriculture Sector
[119,123]	Industrial Control Systems	Method Design	Protecting and maintaining data in industrial control systems to provide a trustworthy data source	Providing trustworthy data sources for the food and agriculture sectors, ensuring the accuracy and reliability of data during the production process
[120,121,125]	Communication Systems	Scheme/Model Design	Preventing data forgery or loss during the communication phase	Protecting data in the supply chain from tampering, ensuring the transparency and security of food traceability and supply chain management
[122,124]	Cloud Storage	Framework Design	Safely recording data operations that occur in the cloud environment	Achieving the secure storage and management of vast agricultural data, such as crop monitoring, soil analysis, and market data, in the cloud, improving the efficiency and security of data processing

**Table 11:** OTCI data backup and recovery.

Literature	Field	Research Method	Effect	Impact on the Food and Agriculture Sector
[127]	Internet of Things	Method Design	BT-based IoT architecture enabling distributed storage	Protecting agricultural data from tampering, enhancing the transparency and traceability of the food supply chain
[128]	Healthcare Systems	Method Design	Data and interaction records distributed on blockchain for trusted recovery of electronic healthcare systems	Ensuring the security of agricultural workers’ health records helps increase trust in the healthcare services provided to agricultural communities
[126,129]	Industrial Control Systems	Method Design	Supporting data recovery after attacks through efficient replication mechanisms	Helping agricultural control systems quickly recover critical data after a cyberattack reduces the potential impact on agricultural production and the food supply chain

**Table 12:** Common types of cyberattacks in OTCI.

Attack Category	Description
Distributed denial of service attack	Attackers send a large number of requests to the target system, occupying its resources, causing the system to fail to run properly and thus interrupting services.
Ransomware attack	Attackers encrypt critical systems or data with malware and then blackmail the injured party into paying a ransom to unlock them.
Supply chain attack	By planting malware or tampering with hardware at key points in the supply chain, attackers can exploit vulnerabilities to attack targeted systems when products or services are delivered to end users.
Malware attack	Attackers can gain improper access or steal sensitive information by introducing malware, such as viruses, trojans, or spyware, into a target system.
Insider attack	Refers to the abuse of access rights by individuals within a business or organization to attack CI or intentionally disclose sensitive information.
Zero-day attack	Exploit undisclosed vulnerabilities in software or systems. These vulnerabilities are often unpatched and can be used by an attacker to break into a system before the attacker is aware of them.
Identity fraud	Attackers use forged identities to impersonate legitimate users and gain access to CI.
Social engineering attack	Attackers gain access to systems or sensitive information by communicating with people or spoofing them, such as phishing emails, phone scams, etc.

**Table 13:** Intelligent protection model based on BT.

Phase	Model	Problem Solving
Pre-Attack stage of OTCI	Blockchain-based data recording model	Identity authentication and verification of equipment, personnel and data, permission distribution and access control, and data recording and leaving marks throughout the process
	Blockchain-based identity authentication model	
	Blockchain-based data verification model	
	Blockchain-based permission distribution model	
	Blockchain-based access control model	
During-the-attack stage of OTCI	Blockchain-based attack detection model	Detection and defense of network attack models
	Blockchain-based data protection model	
Post-attack stage of OTCI	Blockchain-based attack assessment model	Cyberattack analysis, data recovery, and location and accountability

## Data Availability

The authors declare that the data supporting the findings of this study are available from the authors.

## Appendix A

**No.**	**Journal Name**	**TP**	**IF**	**H**	**WQ**	**Subject Area**
1	IEEE INTERNET OF THINGS JOURNAL	9	8.2	47	Q1	COMPUTER SCIENCE
2	IEEE ACCESS	9	3.4	56	Q2	COMPUTER SCIENCE
3	ELECTRONICS	4	2.6	21	Q2	ENGINEERING
4	CLUSTER COMPUTING-THE JOURNAL OF NETWORKS SOFTWARE TOOLS AND APPLICATIONS	3	3.6	35	Q1	COMPUTER SCIENCE
5	WIRELESS PERSONAL COMMUNICATIONS	2	1.9	48	Q3	COMPUTER SCIENCE
6	SUSTAINABLE CITIES AND SOCIETY	2	10.5	25	Q1	ENGINEERING
7	SUSTAINABILITY	2	3.6	53	Q2	ENVIRONMENTAL SCIENCES
8	SCIENTIFIC REPORTS	2	3.8	149	Q1	MULTIDISCIPLINARY SCIENCES
9	JOURNAL OF SYSTEMS ARCHITECTURE	2	3.7	42	Q1	COMPUTER SCIENCE
10	IEEE TRANSACTIONS ON INTELLIGENT TRANSPORTATION SYSTEMS	2	7.9	112	Q1	ENGINEERING
11	IEEE TRANSACTIONS ON INDUSTRIAL INFORMATICS	2	11.7	100	Q1	ENGINEERING
12	COMPUTERS & SECURITY	2	4.8	77	Q1	COMPUTER SCIENCE
13	CMC-COMPUTERS MATERIALS & CONTINUA	2	2	27	Q3	COMPUTER SCIENCE
14	WORLD ELECTRIC VEHICLE JOURNAL	1	2.6	None	Q2	ENGINEERING
15	TRANSACTIONS ON EMERGING TELECOMMUNICATIONS TECHNOLOGIES	1	2.5	18	Q3	TELECOMMUNICATIONS
16	SYMMETRY-BASEL	1	2.2	24	Q2	MATHEMATICS
17	SENSORS	1	3.4	132	Q2	MULTIDISCIPLINARY SCIENCES
18	PERVASIVE AND MOBILE COMPUTING	1	3	53	Q2	COMPUTER SCIENCE
19	NEURAL COMPUTING & APPLICATIONS	1	4.5	57	Q2	COMPUTER SCIENCE
20	KUWAIT JOURNAL OF SCIENCE	1	1.2	9	Q3	MULTIDISCIPLINARY SCIENCES
21	JOURNAL OF THE BRITISH BLOCKCHAIN ASSOCIATION	1	1.4	None	Q3	ECONOMICS
22	JOURNAL OF SUPERCOMPUTING	1	2.5	49	Q2	COMPUTER SCIENCE
23	JOURNAL OF NETWORK AND SYSTEMS MANAGEMENT	1	4.1	30	Q1	COMPUTER SCIENCE
24	JOURNAL OF MEDICAL INTERNET RESEARCH	1	5.8	116	Q1	MEDICAL
25	JOURNAL OF INTELLIGENT & FUZZY SYSTEMS	1	1.7	46	Q3	COMPUTER SCIENCE
26	JOURNAL OF INFORMATION SECURITY AND APPLICATIONS	1	3.8	None	Q2	COMPUTER SCIENCE
27	JOURNAL OF ENTERPRISE INFORMATION MANAGEMENT	1	7.4	None	Q1	MANAGEMENT
28	Journal of Applied Sciences	1	None	None	None	COMPUTER SCIENCE
29	INTERNATIONAL JOURNAL OF DISTRIBUTED SENSOR NETWORKS	1	1.9	38	Q3	COMPUTER SCIENCE
30	INTERNATIONAL JOURNAL OF ADVANCED COMPUTER SCIENCE AND APPLICATIONS	1	0.7	None	Q3	COMPUTER SCIENCE, THEORY & METHODS
31	INTELLIGENT COMPUTING	1	None	None	None	COMPUTER SCIENCE
32	INFORMATION PROCESSING & MANAGEMENT	1	7.4	88	Q1	MANAGEMENT, COMPUTER SCIENCE
33	INFORMATION	1	2.4	None	Q3	COMPUTER SCIENCE, INFORMATION SYSTEMS
34	IEEE WIRELESS COMMUNICATIONS	1	10.9	139	Q1	COMPUTER SCIENCE
35	IEEE TRANSACTIONS ON SERVICES COMPUTING	1	5.5	56	Q1	COMPUTER SCIENCE
36	IEEE TRANSACTIONS ON NETWORK AND SERVICE MANAGEMENT	1	4.7	31	Q1	COMPUTER SCIENCE
37	IEEE TRANSACTIONS ON EMERGING TOPICS IN COMPUTING	1	5.1	31	Q1	COMPUTER SCIENCE
38	IEEE TRANSACTIONS ON DEPENDABLE AND SECURE COMPUTING	1	7	59	Q1	COMPUTER SCIENCE
39	IEEE NETWORK	1	6.8	111	Q1	COMPUTER SCIENCE
40	IEEE COMMUNICATIONS MAGAZINE	1	8.3	213	Q1	COMPUTER SCIENCE
41	FUTURE GENERATION COMPUTER SYSTEMS-THE INTERNATIONAL JOURNAL OF ESCIENCE	1	6.2	93	Q1	COMPUTER SCIENCE
42	DRONES	1	4.4	None	Q1	REMOTE SENSING
43	DIAGNOSTICS	1	3	None	Q1	MEDICINE
44	CONCURRENCY AND COMPUTATION-PRACTICE & EXPERIENCE	1	1.5	60	Q2	COMPUTER SCIENCE
45	COMPUTERS IN INDUSTRY	1	8.2	87	Q1	COMPUTER SCIENCE
46	COMPUTERS & ELECTRICAL ENGINEERING	1	4	49	Q1	COMPUTER SCIENCE
47	COMPUTER-AIDED CIVIL AND INFRASTRUCTURE ENGINEERING	1	8.5	68	Q1	CONSTRUCTION & BUILDING TECHNOLOGY
48	COMPUTER NETWORKS	1	4.4	119	Q1	COMPUTER SCIENCE
49	COMPUTER COMMUNICATIONS	1	4.5	91	Q1	COMPUTER SCIENCE
50	COMPUTATIONAL INTELLIGENCE AND NEUROSCIENCE	1	None	42	None	MATHEMATICAL & COMPUTATIONAL BIOLOGY-NEUROSCIENCES
51	APPLIED ARTIFICIAL INTELLIGENCE	1	2.9	52	Q2	COMPUTER SCIENCE
52	ALEXANDRIA ENGINEERING JOURNAL	1	6.2	None	Q1	ENGINEERING, MULTIDISCIPLINARY
53	AD HOC NETWORKS	1	4.4	79	Q1	COMPUTER SCIENCE
		TP = total publications; H = h-index; IF = impact factor; WQ = WoS quartile.				
